# Transcriptome Analysis of Differentially Expressed Genes Involved in Proanthocyanidin Accumulation in the Rhizomes of *Fagopyrum dibotrys* and an Irradiation-Induced Mutant

**DOI:** 10.3389/fphys.2016.00100

**Published:** 2016-03-18

**Authors:** Caixia Chen, Ailian Li

**Affiliations:** The Cultivation Center, Institute of Medicinal Plant Development, Peking Union Medical College, Chinese Academy of Medical SciencesBeijing, China

**Keywords:** *Fagopyrum dibotrys*, irradiation, RNA-seq, proanthocyanidin accumulation, molecular mechanism

## Abstract

The rhizome of *Fagopyrum dibotrys* is a traditional Chinese medicine that has recently gained attention due to substantial findings regarding its bioactive proanthocyanidin (PA) compounds. However, the molecular mechanism underlying PA accumulation in *F. dibotrys* remains elusive. We previously obtained an irradiation-induced mutant (RM_R) of *F. dibotrys* that had a higher PA content compared to that of the wild-type (CK_R). The present study aimed to elucidate the molecular mechanism underlying PA accumulation in *F. dibotrys* by comparing the rhizome transcriptomes of the irradiation-induced mutant and wild-type using RNA-seq analysis. A total of 53,540 unigenes were obtained, of which 29,901 (55.84%) were annotated based on BLAST searches against public databases, and 501 unique sequences were differentially expressed between the two samples, which consisted of 204 up-regulated and 297 down-regulated unigenes. Further analysis showed that the expression patterns of some unigenes encoding enzymes involved in PAs biosynthesis in *F. dibotr*ys rhizomes differed between RM_R and CK_R. In addition, we identified transcription factor families and several cytochrome P450s that may be involved in PA regulation in *F. dibotrys*. Finally, 12 unigenes that encode PA biosynthetic enzymes were confirmed by qRT-PCR analysis. This study sheds light on the molecular mechanism underlying radiation-mediated flavonoid accumulation and regulation in *F. dibotrys* rhizomes. These results will also provide a platform for further functional genomic research on this particular species.

## Introduction

*Fagopyrum dibotrys* (D. Don) Hara (perennial buckwheat), which belongs to the genus *Fagopyrum* (Mill) of the Polygonaceae family, is an erect perennial herb of significant medicinal and economic values. The rhizome of *F. dibotrys* is commonly used in traditional Chinese medicine for the treatment of lung abscesses, dysentery, rheumatism, throat inflammation, and tumefaction (Editorial Board of Zhong Hua Ben Cao, 1999). In addition, the grains of *F. dibotrys* have higher nutritional value and healthy benefits (Fggum et al., 1980; De Francischi et al., 1994; Guo et al., 2007), and its stem and leaf are also good forage feed resources (Deng et al., 2012). Currently, more attention has been given to this species because of its medicinal value. Modern pharmacological research indicates that the active extract (mainly products of the flavonoid secondary metabolism) of the *F. dibotrys* rhizome is highly effective in anticancer, antidiabetics, regulating blood lipid, antioxidant, analgesic, and antipyretic (Chan, 2003; Wang et al., 2005). Recent studies have shown that multi-hydroxyl tannins in the rhizome of *F. dibotrys* has widespread biological activity such as exerting an anti-neoplastic effect by means of anti-mutagen and inhibiting tumor promoter (Kakiuchi et al., 1985; Okuda et al., 1985; Chen and Gu, 2000; Chen et al., 2005; Zhang et al., 2010). Yao et al. also showed that its bioactive compounds included a mixture of proanthocyanidins (PAs; also known as condensed tannins) whose basic unit is (−)-epicatechin, which has inhibitory effects on tumor cells *in vitro* and in experimental animal tumors (Yao et al., 1989). PAs are one of the final products of the flavonoid pathway and provide multiple health benefits to humans such as antioxidant, anticancer, and anti-cardiovascular effects (Yang and Koo, 2000; Bemis et al., 2006; Luximon-Ramma et al., 2006). The study of anti-tumor compounds and related biosynthetic pathways is thus an important field in buckwheat research.

*F. dibotrys* is one of the national key conserved wild plants of China, as sanctified by the State Council of Traditional Chinese Medicine in 1999. In recent years, the *Fagopyrum* species has attracted wide attention of the pharmaceutical industry both in China and the rest of the world for its active component, the flavonoids (Li et al., 2010). However, its wild resources have dramatically decreased in the past years, and the cultivars of *F. dibotrys* have a limited yield and lower nutrient value and medicinal efficacy than that of the wild-type. (−)-Epicatechin, the end products of the flavonoid secondary metabolism, is the biomarker used by the Chinese pharmacopoeia (Chinese Pharmacopoeia Commission, 2015) in evaluating the quality of *F. dibotrys*. The biosynthesis of flavonoids often increases in response to external stress factors such as drought, cold temperatures, wounding, or excess UV light (Winkel-Shirley, 2002). Previous studies have suggested that irradiation plays a role in stress response by inducing the accumulation of secondary metabolites (Laura et al., 2004; Guan et al., 2006; Jia and Li, 2007). Radiation-induced plant can provide a responsive system for the identification and profiling of transcripts and regulatory factors involved in secondary metabolite accumulation. In our previous study, rhizomes of green-stemmed perennial buckwheat from Jiangsu Province (*CK_R*) were irradiated with gamma rays, and a red-stemmed perennial buckwheat mutant (*RM_R*) was obtained from the M2 generation. Analyses of rhizome extracts by high-performance liquid chromatography (HPLC) have shown that the mutant plant (RM_R) had a higher level of (−)-epicatechin compared to that of the wild-type (CK_R)(Jia and Li, 2008). Further studies proved the stability of morphological and biochemical characteristics [(−)Epicatechin content] of the mutant plants (Chen et al., 2012). Changes in the level of the active compound often result in different pharmacological activity and medicinal quality. Therefore, a systematic study comparing gene expression patterns between the irradiation-induced mutant and the wild-type is of essence.

Much effort has been made in elucidating the biosynthetic pathways of PAs from a genetic perspective. Mutants affecting flavonoid synthesis have been isolated from a wide range of plant species. Maize, snapdragon, and petunia have been established as major experimental models of this system, leading to the isolation of various structural and regulatory flavonoid genes (Maria et al., 2012). In model plants, this PA biosynthetic pathway can be divided into three stages (Himi et al., 2011). The first stage is the conversion of phenylalanine to coumarate-CoA by phenylalanine ammonialyase gene (*PAL*), cinnamate 4-hydroxylase gene (*C4H*), and 4-coumarate CoA ligase gene (*4CL*). The second stage is the formation of dihydroflavonol by one molecule of coumarate-CoA and three molecules of malonyl-CoA, which is catalyzed by the chalcone synthase gene (*CHS*), chalcone isomerase gene (*CHI*), flavanone 3-hydroxylase gene (*F3H*), flavonoid 3′-hydroxylase gene (*F3*′*H*), flavonoid 3′,5′-hydroxylase gene (*F3*′*5*′*H*), and flavonol synthase gene (*FLS*). The third stage is the formation of PAs by dihydroflavonols as catalyzed by the dihydroflavonol 4-reductase gene (*DFR*) and leucoanthocyanidin reductase gene (*LAR*), which converts leucoanthocyanidins to 2,3-cis-flavan-3-ols. PAs are colorless phenolic oligomers that result from the condensation of flavan-3-ol units. PA biosynthesis is composed of synthetic pathways controlled by both structural genes encoding enzymes that directly participate in the biochemical reactions and regulatory genes that control the transcription of structural genes (Lepiniec et al., 2006; Takashi et al., 2009). Some of the enzyme-encoding genes of *F. dibotrys* such as *FdPAL, FdFLS, FdCHI, FdCHS, FdDFR, FdANS, FdLAR*, and *FdMYB* of flavonoid biosynthetic pathways have been cloned and analyzed (Liu et al., 2009; Ma et al., 2009, 2012; Meng et al., 2010; Li et al., 2011, 2012; Jiang et al., 2013; Luo et al., 2013; Pu et al., 2014). However, the molecular mechanism of PA accumulation in *F. dibotrys* roots is still unclear, and research studies on the relationship between the abundance of active compounds and gene expression during PA biosynthesis in *F. dibotrys* using molecular biological techniques are limited.

In recent years, RNA sequencing has revolutionized the exploration of gene expression. RNA sequencing (RNA-seq) provides a fast, cost-effective, and reliable approach to generate large expression datasets for functional genomic analysis, which is especially suitable for non-model species with unsequenced genomes (Shi et al., 2011; Hao et al., 2012; Chen et al., 2014; Noor et al., 2014). In addition, RNA-seq is more suitable for comparative gene expression analysis and generates ultra-high-throughput data that cover numerous low-abundance genes because of its superior sequencing depth and high level of sensitivity (Gutha et al., 2010; Liu et al., 2014; Shi et al., 2014; Wang L. et al., 2014; Guo et al., 2015). Logacheva et al. (2011) performed *de novo* sequencing and characterization of the floral transcriptome of two Polygonaceae, *Fagopyrum esculentum* and *F. tataricum*, using 454 pyrosequencing technology; however, the genes involved in secondary metabolism, as well as molecular markers and repetitive sequences, were not studied. It is well known that plant materials with obvious differences are essential in the study of gene function. Furthermore, genetic mutants that control relative characteristics are the best materials to study gene function. In the present study, the *F. dibotrys* irradiation-mutant (RM_R) that contains a relatively higher level of bioactive compounds and the control plant (CK-R) with a consistent genetic background were used as experimental materials, and Illumina HiSeq 2000 platform sequencing was adopted to analyze differences in active compound accumulation. A total of 82,814,230 high-quality reads were generated, from which 29,901 unigenes were obtained by *de novo* assembly. Genes that may participate in flavonoid biosynthesis of *F. dibotrys* were identified based on annotation and expression levels. The expression patterns of unigenes identified in the PA biosynthetic pathway were validated through quantitative reverse transcription-polymerase chain reaction (qRT-PCR), and quantitative analysis of PAs was performed using HPLC, thereby revealing the different molecular mechanism of PA accumulation in *F. dibotrys* rhizomes. These results provide a foundation for breeding active compounds *F. dibotrys* varieties, and a platform of sequence information for the global discovery of novel functional genes involved in the biosynthesis of PA in *F. dibotrys*.

## Materials and methods

### Plant materials and treatment condition

**Figure 1:**
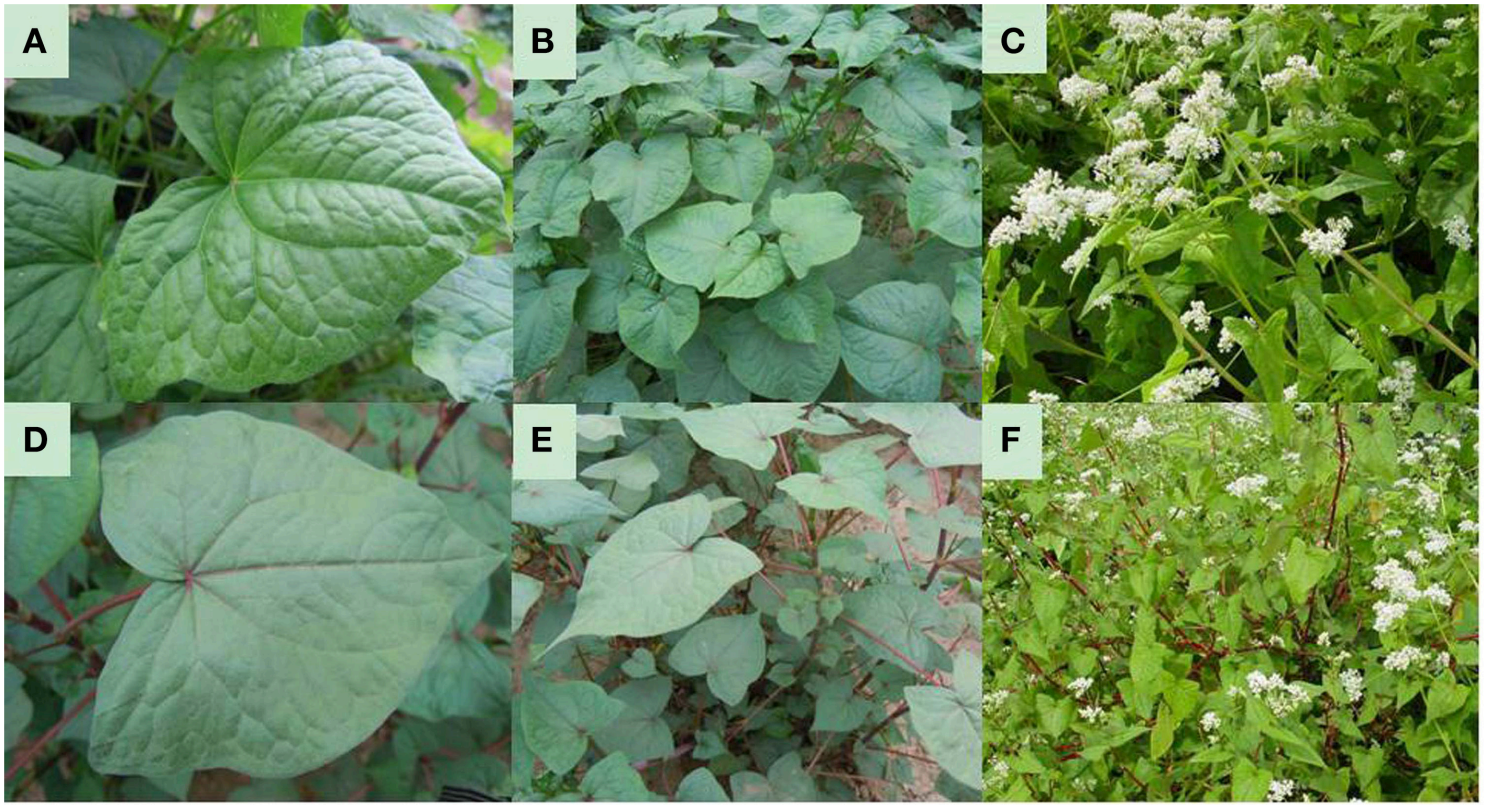
**Plant materials. (A–C)**, CK_R plant: **(A)**, CK_R leaves, veins and petioles are indicated in green; **(B)**, CK_R plant, stems are indicated in green; numerous primary branches were observed; **(C)**, CK_R plant bloom. **(D–F)**, RM_R Plant: **(D)**, RM_R leaves, veins and petioles are indicated in red; **(E)**, RM_R Plant, stems are depicted in red; a lower number of primary branches were observed; **(F)**, RM_R Plant in bloom.

The wild-type of *F. dibotrys* (CK_R) plants from Jiangsu province were authenticated by Professor Ailian Li of the Institute of Medicinal Plant Development (IMPLAD) using the morphological identification approach of the Flora of China. The *F. dibotrys* mutant (RM_R) was introduced by means of γCo^60^ irradiation on the rhizomes of the CK_R plant in 2007 and then screened in the M_2_ generation. The RM_R plants were reproduced asexually and had better stability of characters, as described in our previous report (Jia and Li, 2008; Lan et al., 2011; Chen et al., 2012). The RM_R and CK_R plants were used as test materials for comparative analysis in the present study (Figure 1). These plants were cultured at the cultivation and experiment base of IMPLAD (lat. 39°47′ N, long. 116°25′ E, alt. 50 m), which is located in a temperate continental climatic region.

The roots of 1-year-old plants were collected in early November, which is the routine harvest time of medicinal materials from *F. dibotrys*. After rinsing in distilled water, the roots were then gently dried and quickly dabbed in absorbent paper, cut into small pieces, flash-frozen in liquid nitrogen, and stored at −80°C until RNA isolation. Meanwhile, the roots of three individual RM_R and CK_R plants were separately harvested and stored for active compound detection and qRT-PCR analysis.

### RNA isolation and library preparation

Total RNA was isolated from the roots using the RNeasy plant kit (BioTeke, Beijing, China). RNA degradation and contamination were monitored using EtBr-stained 1% agarose gels, and RNA purity was analyzed using a NanoPhotometer® spectrophotometer (IMPLEN, Westlake Village, CA, USA). RNA concentration was assessed using a Qubit RNA Assay Kit in Qubit ® 2.0 Fluorometer (Life Technologies, Foster City, CA, USA), and RNA integrity was assessed using an RNA Nano 6000 Assay Kit of an Bioanalyzer 2100 system (Agilent Technologies, Santa Clara, CA, USA).

A total amount of 3 μg of RNA per sample was used as input material in the RNA analysis. The two samples, including CK_R as control and RM_R as treatment, had RNA integrity number (RIN) values above 8. The sequencing libraries were generated using IlluminaTruSeq™ RNA Sample Preparation Kit (Illumina, San Diego, CA, USA) by following manufacturer's recommendations, and index codes were added to attribute the sequences to each sample. Briefly, mRNA was purified from total RNA using poly-T oligo-attached magnetic beads. Fragmentation was performed using divalent cations under an elevated temperature in an Illumina proprietary fragmentation buffer. First-strand cDNA was synthesized using random oligonucleotides and SuperScript II (Invitrogen, Carlsbad, CA, USA). cDNA synthesis was subsequently performed using DNA Polymerase I and RNase H. The remaining overhangs were converted into blunt ends via exonuclease/polymerase activities, and the enzymes were removed. After adenylation of the 3′ ends of the DNA fragments, Illumina paired-end adapter oligonucleotides were ligated to prepare for hybridization. The library fragments were purified with in an AMPure XP system (Beckman Coulter, Beverly, MA, USA). The DNA fragments with the ligated adaptor molecules on both ends were selectively enriched using an Illumina PCR Primer Cocktail in a 10-cycle PCR reaction. The products were purified (AMPure XP system) and quantified using the Agilent high-sensitivity DNA assay on an Agilent Bioanalyzer2100 system.

Clustering of the index-coded samples was performed on a cBot Cluster Generation System using TruSeq PE Cluster Kit v3-cBot-HS (Illumina), according to the manufacturer's instructions. After cluster generation, the library preparations were then sequenced on an Illumina HiSeq 2000 platform and 90-bp paired-end reads were generated. All reads were deposited in the Short Read Archive (SRA) of the National Center for Biotechnology Information (NCBI) public database, with the accession number PRJNA309922.

### Data processing, assembly, and annotation

The raw data (raw reads) of the fastq format was processed by removing the reads that contained adapters and poly-Ns, as well as low-quality reads to generate clean data (clean reads). Here, the low-quality reads are that the number of bases which Q-score is less than or equal to 5 accounts for more than 50% of the entire read. The Q20, Q30, and GC content and sequence duplication level of the clean data were simultaneously calculated. All downstream analyses were based on clean data with high quality. The left-end files (read1 files) from the two libraries/samples were pooled into one big left.fq file, and the right-end files (read2 files) into one big right.fq file. Transcriptome assembly was accomplished based on the left.fq and right.fq using Trinity (Grabherr et al., 2011) with min_kmer_cov set to 2, and all other parameters set to default.

Gene function was annotated based on the following databases: NCBI non-redundant protein sequences (Nr), NCBI non-redundant nucleotide sequences (Nt), protein family (Pfam), a manually annotated and reviewed protein sequence database (Swiss-Prot), KEGG Ortholog database (KO), and Gene Ontology (GO). We used the NCBI blast 2.2.28+ to make the NR, NT, SwissProt and KOG annotation; The HMMER 3.0 package is used to PFAM annotation. The Blast 2 GO v2.5 is used to GO function annotation according to the NR and Pfam protein annotation; KEGG Automatic Annotation Server is used to KEGG annotation.

Gene expression levels were estimated by RSEM (Li and Dewey, 2011) for each sample. First, the clean data were mapped back onto the assembled transcriptome. Second, the read count for each gene was obtained from the mapping results.

### Differential expression analysis

Prior to differential gene expression analysis, for each sequenced library, the read counts were adjusted by using the edgeR program package through one scaling normalized factor. Differential expression analysis of two samples was performed using the DEGseq (Wang et al., 2010) R package. *P*-values were adjusted using the *Q*-value (Storey and Tibshirani, 2003). The cut off value of *Q* < 0.005 and at least 2 fold change were set as the threshold for differential expression.

The identified DEGs expression were using K-means method and then were used for further GO and KO enrichment analysis. GO enrichment analysis of the differentially expressed genes (DEGs) was implemented by the GOseq R packages based Wallenius non-central hyper-geometric distribution (Young et al., 2010), which can adjust for gene length bias in DEGs. KEGG (Kanehisa et al., 2008) is a database resource for understanding high-level functions and utilities of the biological system such as the cell, the organism, and the ecosystem, from molecular-level information, especially large-scale molecular datasets generated by genome sequencing and other high-throughput experimental technologies (http://www.genome.jp/kegg/). We used the KOBAS (Mao et al., 2005) software to test the statistical enrichment of DEGs in the KEGG pathways.

### Analysis of epicatechin and catechin by HPLC

Chemical analysis of epicatechin and catechin was conducted by HPLC analysis and a minor modification of a previously published method (Kim et al., 2014). Briefly, dried samples of *F. dibotrys* rhizomes were ground into a fine powder using a grinder. Powdered samples (~150 mg) were subjected to extraction via ultra-sonication with 80% (v/v) methanol at room temperature for 60 min. Subsequently, the extracts were centrifuged, and the supernatant was filtered through a 0.45-μm micromembrane for HPLC analysis. A Merck C18 column (250 mm × 4.6 mm × 5 μm) was used as the stationary phase, and a solution of ultrapure water (pH was adjusted to 3.00 ± 0.02 with phosphoric acid) and acetonitrile (90:10) was the mobile phase, and the flow rate was maintained at 1.0 mL·min^−1^ at 35°C. An injection volume of 20 μL and a wavelength of 280 nm were used for detection. The compounds in the sample were determined using a standard curve. All samples were analyzed in triplicate.

### Real-time quantitative PCR analyses

Twelve unigenes that may be involved in the biosynthesis of flavonoid metabolites were selected for expression profiling using the roots of RM_R and CK_R plants. The unigenes and their putative encoding enzymes are listed in Additional file 1. New plant materials from the individuals were used for the RNA extraction for the qRT-PCR, and three biological replicates were made. *Histone H3* was chosen as the internal reference gene. qRT-PCR analysis was performed with an ABI7500 Real-Time PCR Detection System (Applied Biosystems, Foster City, CA, USA) using a GoTaq® qPCRMasterMix (Promega, Beijing, China), and repeated three times. Total RNA was extracted from *F. dibotrys* roots treated with RNase-free DNase (TaKaRa). Reverse transcription was performed using GoScript™ Reverse Transcription System (Promega), following the manufacturer's recommendations. Each reaction contained 7.5 μL of 2 × GoTaq® qPCR Master Mix (Promega), 10 ng of cDNA sample, and 200 n*M* of gene-specific primers. The total volume of each reaction was 15 μL. The cycling conditions were as follows: 95°C for 2 min, followed by 40 cycles of 95°C for 15 s and then 60°C for 60 s. A melting-curve program was then conducted at a temperature range of 55–85°C, with a 5 s hold at each temperature. PCR amplification was performed using specific primers for the genes listed in Additional file 1. The suitability of the oligo nucleotide sequences in terms of annealing efficiency was evaluated in advance using the PRIMER 3.0 program (Rozen and Skaletsky, 2000). The mean value of three replicates was normalized using *histone H3* as the internal control. Relative expression levels were calculated by comparing the cycle thresholds (CTs) of the target genes with that of the housekeeping gene *histone H3* using the 2−ΔΔCT method (Livak and Schmittgen, 2001).

## Results

### The roots of RM_R AND CK_R have significantly different amounts of (−)-epicatechin and catechin

To determine whether the PA content of RM_R was significantly higher than that of CK_R again and to justify that this was the appropriate test material for comprehensive characterization of the PA accumulation mechanism, HPLC analysis of the PAs [especially (−)-epicatechin and catechin] in the roots of RM_R and CK_R plants was performed. The RM_R plants showed relatively higher PAs, with 6.04 mg·L^−1^(−)-Epicatechin and 14.25 mg·L^−1^ catechin, whereas CK_R had 2.79 mg·L^−1^(−)-epicatechin and 6.08 mg·L^−1^ catechin (Table 1). We assessed the gene expression levels of (−)-epicatechin and catechin biosynthesis in the two samples to provide a more comprehensive overview of flavonoid-associated gene profiles in plants with different flavonoids expression patterns.

**Table 1:** **Comparison of (−)-epicatechin and catechin levels in RM_R and CK_R roots**.

**Sample**	**(−)-Epicatechin(mg.L^−1^)**	**Catechin(mg.L^−1^)**
CK_R	2.79 ± 0.214 a	6.08 ± 0.198 a
RM_R	6.04 ± 0.318 b	14.25 ± 0.567 b

Values are expressed as the mean ± standard error (SE) of three replicates. Values within a column followed by different letters are significantly different at α = 0.05.

### RNA-sequencing and *de novo* assembly

To comprehensively examine the genes associated with flavonoid formation and accumulation, we performed RNA-seq analysis of RM-R and CK-R. Total RNA was extracted from 1-year-old roots of the two plants to develop cDNA libraries. A total of 85,861,012 and 74,191,882 reads were generated from the RM-R and CK-R plants, respectively (Table 2). To ensure the reliability of the libraries, the reads that contained adapter or poly-N and were of low quality were removed from raw data, leaving approximately 82,814,230 and 71,549,604 clean reads for RM-R and CK-R, respectively. Due to the absence of reference genomic sequences, *de novo* assembly was applied to construct transcripts from these RNA-seq reads. In the present study, we used the Trinity (version: v2012-10-05) software for *de novo* assembly of the Illumina reads, which has been demonstrated to be efficient for *de novo* reconstruction of transcriptomes from RNA-seq data (Ilut et al., 2012). The Q20 percentage (percentage of bases whose quality was >20 in clean reads) and GC percent are presented in Table 2. The reads from the two samples were pooled together for more comprehensive reconstruction of transcripts, and a total of 117,191 contigs were obtained from the pool of clean reads, with a mean length of 1308 and length ranging from 201 to 11,410 bp (Table 3). Among the 117,191 contigs, 36,704 unique sequences had lengths within the range of 200–500 bp, 22,760 unique sequences with lengths within the range of 500–1000 bp, and 31,842 unique sequences with lengths within the range of 1000–2000 bp, and 25,885 unique sequences longer than 2000 bp in length (Table 3). The varying lengths suggest that most of the unique sequences (80,487 unique sequences at 68.7%) were longer than 500 bp, which could provide enough sequence length for accurate annotation. A total of 53,540 unigenes were obtained.

**Table 2:** **Summary of the statistics of RNA-seq data of *F. dibotrys* libraries of RM-R and CK-R plant roots**.

**Sample**	**No. of raw reads**	**No. of clean reads**	**Clean bases**	**Error (%)**	**Q20 (%)**	**GC (%)**
RM_R1	42,930,506	41,407,115	4.14Gb	0.03	97.35	45.42
RM_R2	42,930,506	41,407,115	4.14Gb	0.03	96.89	45.46
CK_R1	37,095,941	35,774,802	3.58Gb	0.03	97.29	45.04
CK_R2	37,095,941	35,774,802	3.58Gb	0.03	96.84	45.10

We used an Illumina HiSeq 2000 platform to sequence the transcriptome of the two samples and 90-bp paired-end (named as R1 and R2 for the paired-end data) reads were generated for these cDNA libraries.

**Table 3:** **Summary of *de novo* assembly quality of *F. dibotrys* RNA-seq data**.

**ASSEMBLY QUALITY PARAMETERS**	
Contigs generated	117,191
Maximum contig length	11,410
Minimum contig length	201
Average contig length	1308
Contigs 200–500 bp	36,704
Contigs 500–1 kb	22,760
Contigs 1–2 Kb	31,842
Contigs ≥ 2 Kb	25,885
N50-value	2040
N90-value	625

### Unique sequence annotation

Unique sequence annotation was performed using BLAST analysis (*E* ≤ 10^−5^) against Nr, Nt, KO, Swiss-Prot, PFAM, GO, and KOG databases. A total of 27,626 unigenes (51.59% of all unigenes) were annotated with a significant BLAST result in the Nr database, 20,684 unigenes (38.63% of all unigenes) were annotated in the Swiss-Prot database, 20,114 unigenes were annotated in PFAM, and 22,537 unigenes in GO. A total of 29,901 unigenes (55.84% of all unigenes) were annotated in seven databases (Table 4).

**Table 4:** **Gene annotation results by searching against public databases**.

	**Number of unigenes**	**(%)**
Annotated in NR	27,626	51.59
Annotated in NT	10,890	20.33
Annotated in KO	9301	17.37
Annotated in SwissProt	20,684	38.63
Annotated in PFAM	20,114	37.56
Annotated in GO	22,357	41.75
Annotated in KOG	11,411	21.31
Annotated in all databases	3819	7.13
Annotated in at least one Database	29,901	55.84
Total unigenes	53,540	100

### GO classification

**Figure 2:**
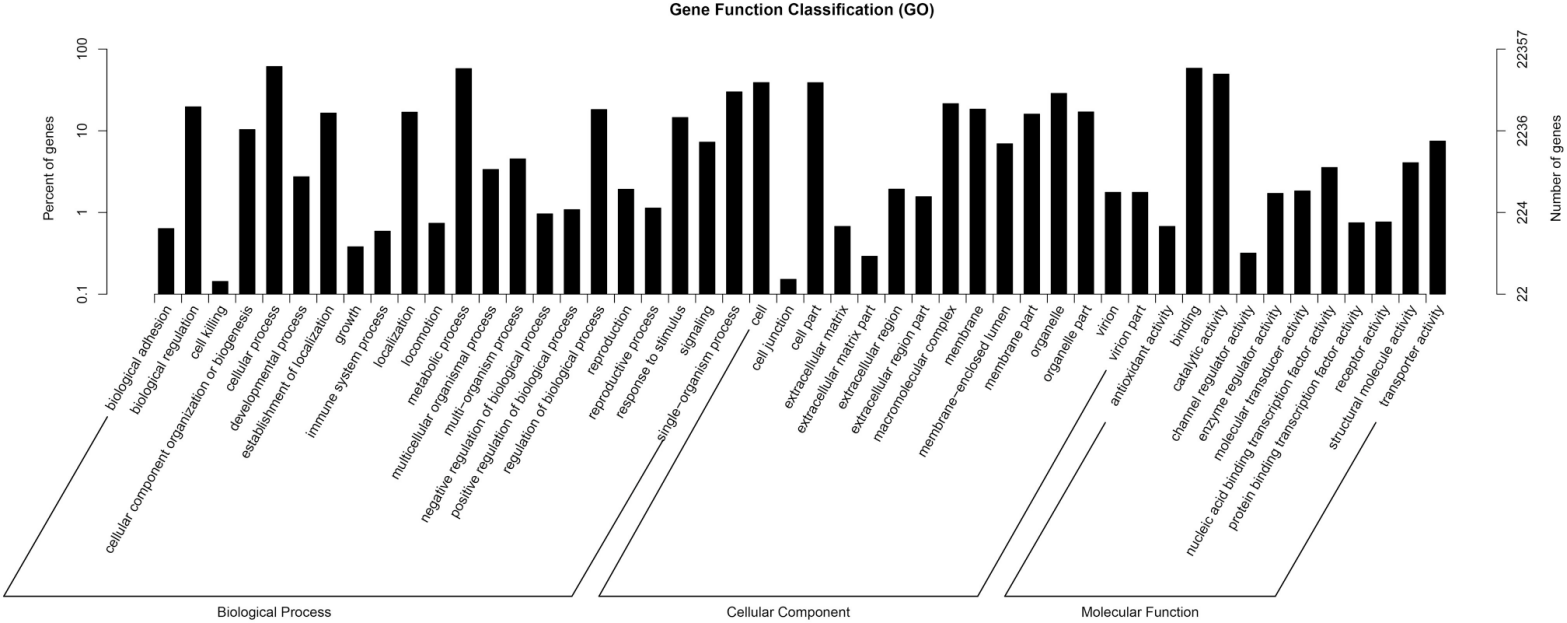
**Histogram of gene ontology classification**. The results are summarized in three main categories: biological process, cellular component, and molecular function.

GO assignments were used to predict the functions of *F. dibotrys* unigenes by classifying these into various biological processes (Ashburner et al., 2000). The GO terms of three categories, namely, biological process, cellar component, and molecular function, were assigned to categorize the function of the predicted unique sequences. Based on sequence homology, the 22,357 unigenes annotated in the GO database were categorized into 55 functional groups. Among these groups, “cellular process” (GO:0009987), “single-organism process” (GO:0044699), and “metabolic process” (GO:0008152) were dominant within the “biological process” category; “cell” (GO:0005623), “cell part” (GO:0044464), and “organelle” (GO:0043226) categories were dominant in the “cellular component” category; and “binding” (GO:0005488), and “catalytic activity” (GO:0003824) predominated the molecular function category. In addition, we noted that several genes were classified into the “biological regulation,” “regulation of biological process,” “membrane,” and “macromolecular complex” categories, whereas a few genes were classified into the “cell killing” and “cell junction” groups (Figure 2, and Additional file 2).

The main flavonoids in *F. dibotrys* roots include a condensation tannin mixture of proanthocyanins, which are also considered metabolic products. Therefore, we hypothesized that the 12,905 unigenes classified into the “metabolic process” group and the 11,043 unigenes classified into the “catalytic activity” group might serve as good candidates for the identification of key genes that participated in PA biosynthesis and accumulation pathways of *F. dibotrys* (Figure 2, and Additional file 2).

### Functional classification by KEGG

**Figure 3:**
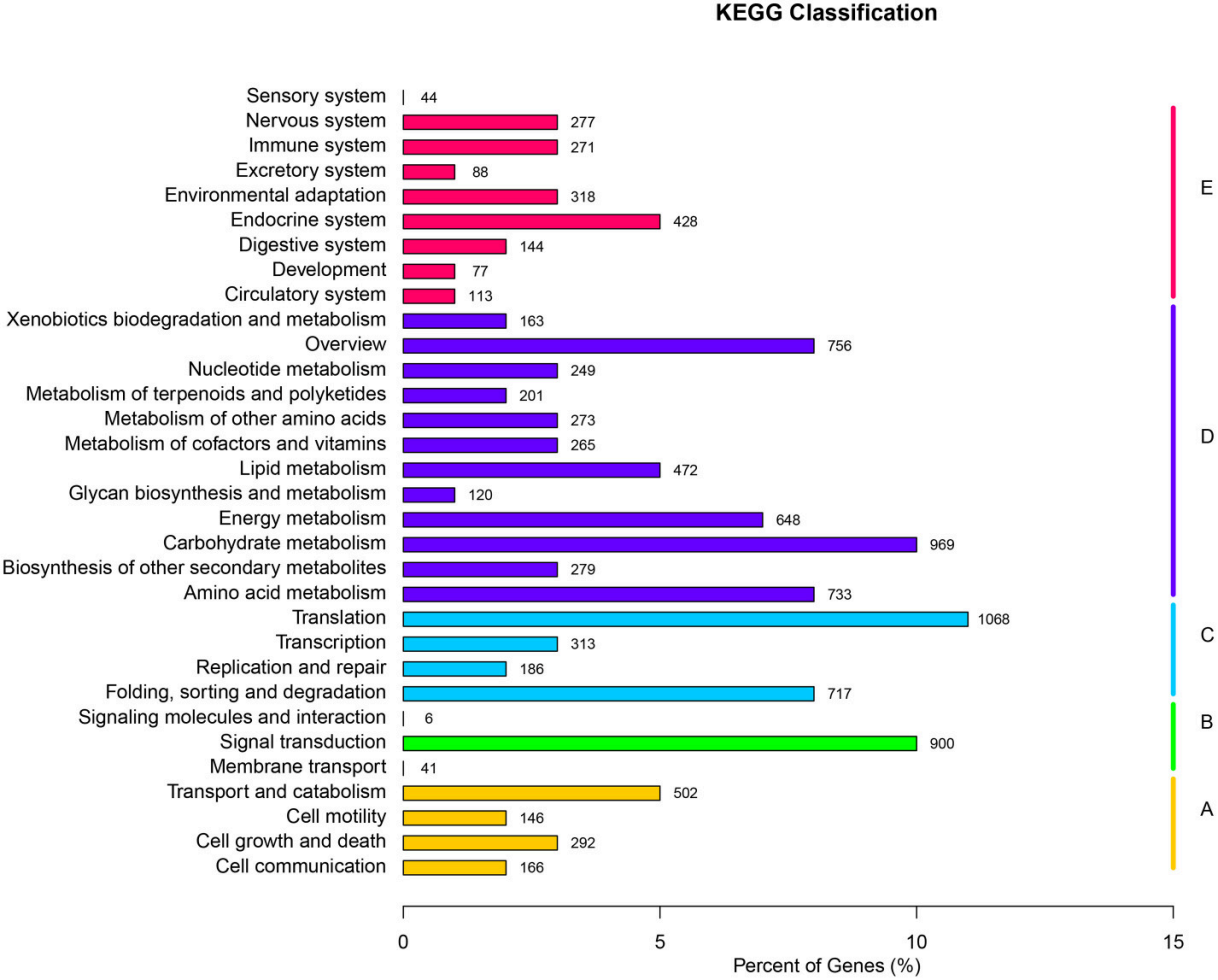
**KEGG classification of all unique sequences. (A)** Cellular processes; **(B)** Environmental information processing; **(C)** Genetic information processing; **(D)** Metabolism; and **(E)** Organismal systems. A total of 9301 unique sequences were classified in the KEGG database.

KEGG serves as a basic platform for the systematic analysis of gene function in terms of the networks of gene products (Kanehisa et al., 2012). To further identify the biosynthesis pathways that are active in *F. dibotrys*, the 9301 unigenes annotated by BLAST analysis against KEGG Automatic Annotation Server (KAAS) were mapped to 261 reference canonical pathways, and these pathways were classified into five main categories: “cellular processes,” “environmental information processing,” “genetic information processing,” “metabolism,” and “organismal systems.” The pathways with the highest representation were “translation” (1068 unigenes, 11.48%), “carbohydrate metabolism” (969 unigenes, 10.42%), and “signal transduction” (900 unigenes, 9.68%) (Figure 3, Additional file 3).

These annotations and classifications may serve as a resource for investigating specific pathways in *F. dibotrys* such as PA biosynthetic pathway. PAs are phenolic oligomers or polymers of flavan-3-ol units. These are synthesized from the first metabolites via shikimate and one of the final products of the flavonoid pathway (Dixon et al., 2005). Therefore, the 279 unigenes clustered into “biosynthesis of other secondary metabolites” might potentially be involved in the biosynthesis and metabolism of flavonoids in *F. dibotrys*. Among the 279 unigenes, 178 unigenes (63.8%), 52 unigenes (18.64%), 14 unigenes (5.02%), and 2 unigenes (0.7%) were classified into the “phenylpropanoid biosynthesis,” “flavonoid biosynthesis,” “flavone, flavonol biosynthesis,” and “anthocyanin biosynthesis” sub-pathways, respectively, and thus were more likely to be involved in PA biosynthesis in the perennial buckwheat (Additional file 3). The annotation of these unique sequences illustrated the transcriptional profile and biochemical pathway of *F. dibotrys*. In addition, these results may serve as a valuable resource for investigating the pathways involved in active compound formation and its accumulation in *F. dibotrys* roots.

### Transcript profiling

**Figure 4:**
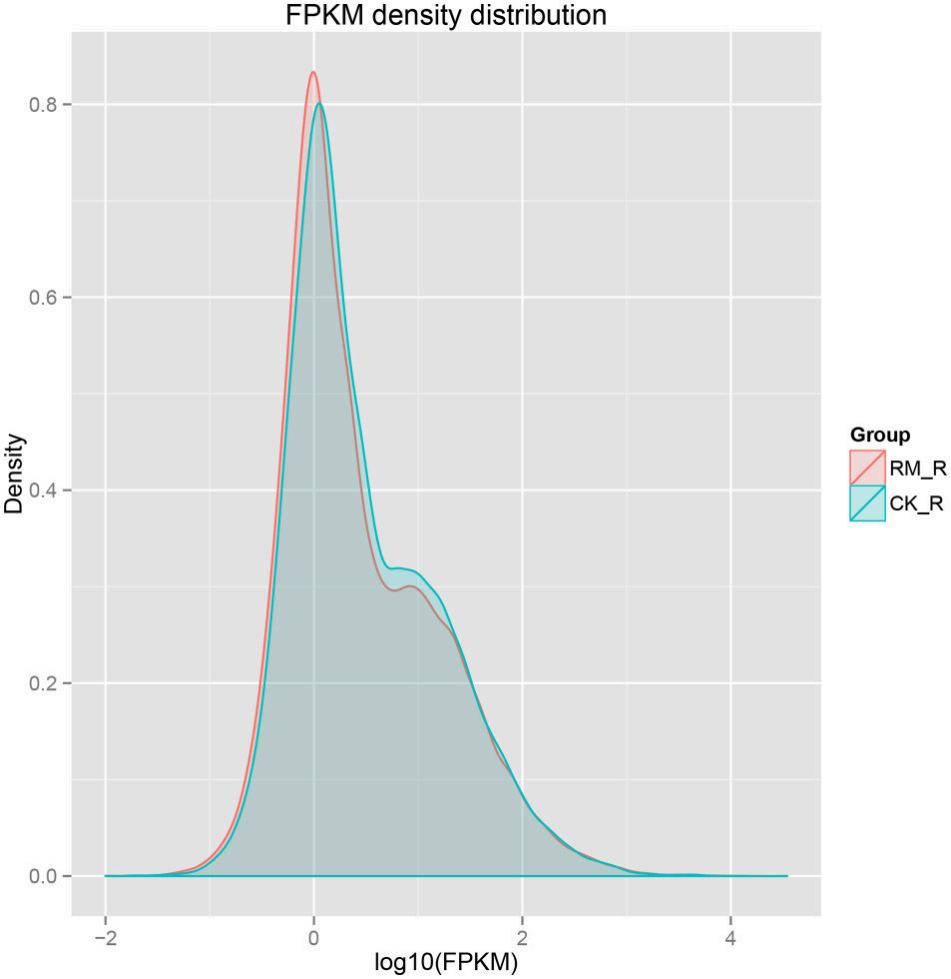
**Frequency distribution of RM-R and CK-R by reads per kilobase per million (RPKM)**.

The sensitivity of RNA-seq facilitates in the measurement of both molar concentration and transcript length. The reads per kilobases per million mapped reads (RPKM) method was used to calculate gene expression levels. We used normalized-RPKM to quantify the transcript level in reads, which facilitated the comparison of mRNA levels both within and among samples (Mortazavi et al., 2008). The RM_R and CK_R plants showed similar RPKM density distributions, which suggested that the transcript profiles were enriched at the RPKM region between 0.3 and 3.57, respectively, and the percentages of transcripts in this region were 53.95 and 52.43%, respectively (Figure 4).

We identified the most highly expressed unigenes in *F. dibotrys* root because these were considered important for *F. dibotrys* development. We focused on the top 100 unigenes with the highest levels of gene expression. The RPKM of those unigenes were >869.2 and 808.8 in RM_R and CK_R plants, respectively. Interestingly, the up-regulated unigenes in the two samples were enriched in biosynthesis and metabolic pathways, as indicated by the results of KEGG and GO analyses (Additional file 4). To date, the root transcriptomes of at least 10 medicinal plants were sequenced and analyzed (Misra, 2014). Although diverse functionally annotated genes were most highly represented in the root transcriptomes of different plants, those genes related to “response to stimulus,” or “response/defense to stress” were generally up-regulated (Sun et al., 2010; Gordo et al., 2012; Sui et al., 2015). This high level of expression was suggestive of an ongoing coping strategy of plant roots to adapt in its immediate soil environment. However, additional evidence is necessary to determine whether the abundance of stress-related genes in the root transcriptome was conserved in *F. dibotrys* or was represented a direct response to its specific conditions. Approximately 10 unigenes were annotated as responding to stimulus/stress in the dataset of RM_R plants, whereas only 6 unigenes were detected in the CK_R plants. The unigenes annotated as chalcone synthase (*CHS*) and chalcone flavonone isomerase (*CHI*), which are involved in flavonoid biosynthesis, were highly expressed in both RM_R and CK_R, in which the RPKM was 1454.4 and 1922.42, and 1237.24 and 1210.97, respectively. On the other hand, the unigene annotated as phenylalanine ammonia-lyase (*PAL*), which is the first key enzyme involved in flavonoid biosynthesis, was most highly expressed (RPKM: 1192.79) in RM_R plants, whereas this was not in the top 100 unigenes that were highly expressed in CK_R plants. In addition, unigenes annotated as transcription factor complexes such as CBF-like transcription factor, WRKY transcription factor, and ethylene-responsive transcription factor ERF 109 were determined to be among the top 100 expressed genes in the RM_R plants, whereas the ethylene-responsive transcription factor ERF 109 and RAP 2-3 were among the top 100 expressed genes in CK_R plants (Additional file 4). These comparisons indicated that genes related to flavonoid metabolisms were differentially expressed in RM_R and CK_M plants.

### Analysis of DEGs

We calculated the gene expression levels based on unique read counts and RPKM values of each unigene to identify the up-regulated and down-regulated genes in the RM_R and CK_R plants. However, to distinguish between significant and non-significant DEGs, additional equations were employed. Differential expression analysis of these two samples was performed using the DEGseq (2010) R package. *P*-value was adjusted using *Q*-value (Storey and Tibshirani, 2003). The cut off value of *Q* < 0.005 and at least 2 fold change were set as the threshold for differential expression.

**Figure 5:**
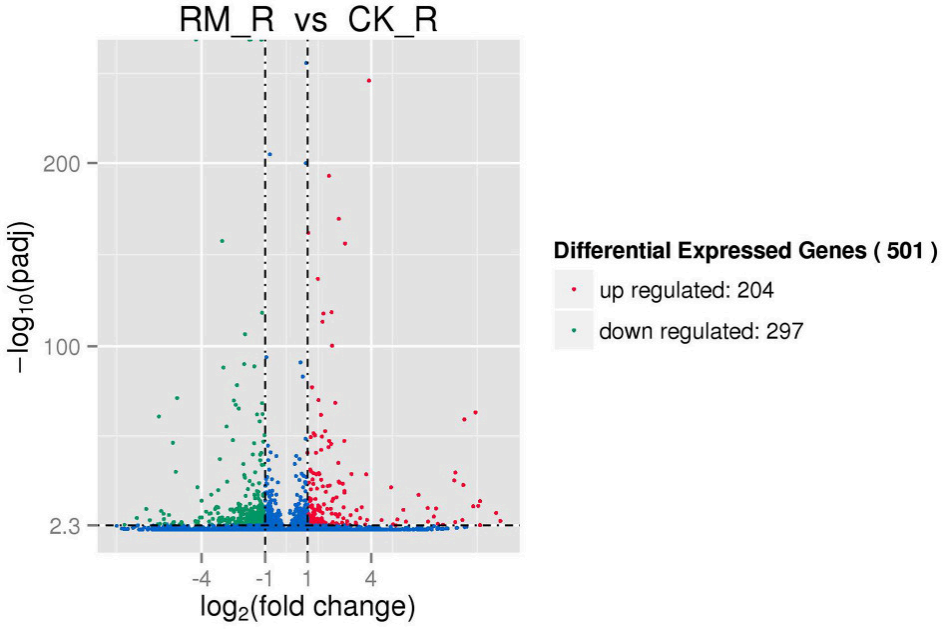
**Differentially expressed genes in two samples of *F. dobotrys***. The significance of different expressed genes should meet to both the absolute value of log_2_. Fold_change. > 1 and *Q* < 0.005.

The RM_R and CK_R plants with significantly different amounts of PAs (epicatechins and catechins) in its roots (Table 1) showed relatively similar transcript profiles (Figure 4). Consequently, we sought to analyze the differentially expressed unigenes to identify candidate genes involved in PA biosynthesis. For RM_R vs. CK_R, 501 unigenes were differentially expressed, including 204 genes that were clearly up-regulated and 297 genes that were markedly down-regulated (Figure 5, Additional file 5).

To further analyze the possible function of unigenes showing differential expression between the two samples, we assessed their GO classifications, as shown in Additional file 6. The DEGs predominantly belonged to “cellular processes” (238), “metabolic processes” (231), “binding” (216), “catalytic activity” (208), “organic substance metabolic process” (186), and “cellular metabolic process” (178). The unigenes showing differential expression between the RM_R and CK_R plants were classified into 82 pathways based on the results of KEGG analysis, with clear enrichment in metabolic pathways (Additional file 7). Of these 82 pathways, the genes involved in metabolic pathways (47 DEGs) and biosynthesis of secondary metabolites (33 DEGs) were predominantly enriched, which indicated that some of the DEGs were likely to be directly or indirectly involved in PA biosynthesis in *F. dibotrys*.

### The expression pattern of genes involved in the PA biosynthetic pathway

**Figure 6:**
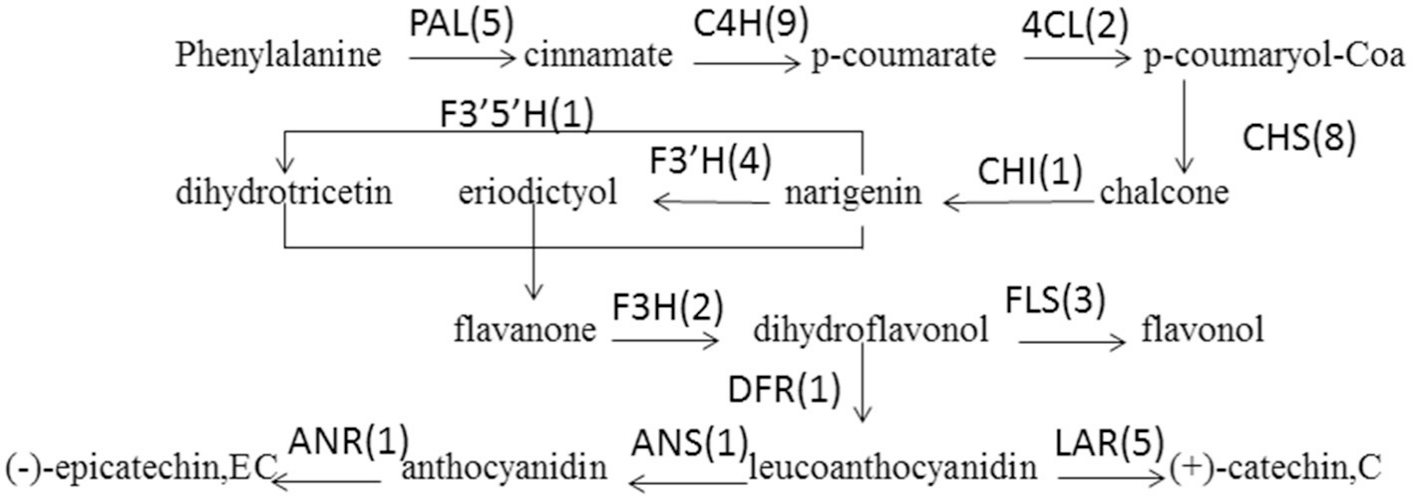
***F. dibotrys* unigenes involved in flavonoid biosynthesis**. The number in bracket following each gene name indicates the number of corresponding *F. dibotrys* unigenes.

PAs are components of metabolites that are synthesized through the general flavonoid pathway. Winkel-Shirley (2002) reported that PAs are derived from the pathway leading to anthocyanins, a class of flavonoids that have been well-characterized at both the biochemical and molecular genetic levels. PA biosynthesis is composed of synthetic pathways controlled by both structural genes encoding enzymes that directly participate in the biochemical reactions and regulatory genes that control the transcription of structural genes (Lepiniec et al., 2006; Takashi et al., 2009). some of the enzyme-encoding genes of *F. dibotrys* such as *FdPAL, FdFLS, FdCHI, FdCHS, FdDFR, FdANS, FdLAR*, and *FdMYB* of flavonoid biosynthetic pathways have been cloned and analyzed. In the present study, all genes involved in PA biosynthesis that were identified in previous study were also discovered and expressed in the current RNA-seq dataset, which was suggestive of the completeness of our transcriptome data. The deduced base PA biosynthetic pathway in *F. dibotrys* roots is presented in Figure 6 (Table 5). The number in the bracket following each gene name indicates the number of corresponding *F. dibotrys* unigenes. Five unigenes of comp40695_c0, comp40849_c1, comp41942_c0, and comp42986_c0 were detected in the *PAL* gene, whereas one unigene had two alternative splicing isoforms, which consisted of comp7222_c0 and comp7222_c1, comp27577_c0 and comp27577_c1, comp31227_c0 and comp31227_c1, and comp29969_c0 and comp29969_c1. The above descriptions indicated that vertical pathway was responsible for the formation and conversion of the subcategories of flavonoids. It is also worth mentioning that flavonoid biosynthesis includes multiple, parallel sub-pathways, which result from flavonoid 3′-hydroxylase and flavonoid 3′,5′-hydroxylase that also accepting the flavones, dihydroflavonols, and flavonols as substrates. Therefore, flavonoid biosynthesis in *F. dibotrys* looks more like a complex metabolic grid than a linear pathway.

**Table 5:** **Expression profiles of genes involved in the biosynthesis of flavonoid in *F. dibotrys* roots**.

**Gene name**	**Length (bp)**	***E*-value**	**RPKM_CK_R**	**RPKM_RM_R**
**PHENYLALANINE AMMONIA-LYASE**				
comp30906_c0	653	1.51E-75	6.63	6.13
comp40695_c0	2537	0	544.95	1192.79
comp40849_c1	3240	0	43.12	42.05
comp41942_c0	941	3.38E-155	38.14	87.09
comp42986_c0	373	7.21E-38	33.38	52.03
**CINNAMIC ACID 4-HYDROXYLASE**				
comp37714_c0	1871	0	33.9	28.52
comp38029_c0	1916	0	282.21	246.94
comp38498_c0	552	1.27E-22	7.29	13.32
comp70348_c0	371	7.15E-63	4.66	8.09
comp7222_c0	254	3.11E-45	6.29	14.61
comp7222_c1	517	3.43E-111	6.42	16.82
comp25500_c0	720	6.79E-106	0.92	1.18
comp26023_c0	1155	1.69E-130	2.5	2.41
comp30892_c0	1916	0	272.6	506.11
**4-COUMARATE:COA LIGASE**				
comp43080_c2	2266	0	34.17	52.82
comp30713_c0	2137	0	20.36	46.56
**FLAVANONE 3′,5′-HYDROXYLASE**				
comp32658_c0	1732	0	2.5	4.61
**FLAVONOID 3′-HYDROXYLASE**				
comp38797_c0	2194	0	77.25	104.74
comp494212_c0	397	4.60E-42	0.14	1.47
comp753300_c0	221	2.32E-26	0	0.97
comp24591_c0	923	1.86E-66	0.23	0.72
**FLAVANONE-3-HYDROXYLASE**				
comp30439_c0	1470	0	176.191	282.2
comp44775_c0	1678	0	283.38	335.13
**CHALCONEISOMERASE**				
comp35337_c0	1283	1.33E-161	116.04	178.11
**CHALCONE SYNTHASE**				
comp19477_c0	1337	0	1.86	2.59
comp27483_c0	689	5.47E-109	0.63	0.53
comp27577_c0	352	1.56E-41	2.88	0.85
comp27577_c1	290	5.04E-51	2.94	2.44
comp2823_c0	207	2.57E-36	2.15	1.34
comp32570_c0	616	3.92E-89	2.88	2.33
comp40750_c0	1742	0	1237.24	1454.4
comp43320_c0	1488	0	78.69	66.48
**FLAVONOL SYNTHASE**				
comp27597_c0	1290	0	7.96	6.4
comp31227_c0	320	1.85E-47	4.28	1.6
comp31227_c1	725	1.86E-129	3.84	1.29
**DIHYDROFLAVONOL-4-REDUCTASE**				
comp29690_c0	1255	0	510.34	718.6
**ANTHOCYANIDIN SYNTHASE**				
comp30968_c0	1498	0	267.53	365.96
**ANTHOCYANIDIN REDUCTASE**				
comp44789_c0	1612	0	276.07	458.4
**LEUCOANTHOCYANTIN REDUCTASE**				
comp2146_c0	591	1.28E-55	0.79	2.65
comp276280_c0	609	9.62E-100	0.41	1.03
comp29969_c0	1001	1.22E-129	81.4	207
comp29969_c1	469	1.24E-17	64.55	225.57
comp35199_c0	1576	0	288	339.64
**CYTOCHROME P450**				
comp35679_c0	1815	0	31.55	83.17

We further investigated the expression levels of genes involved in the PA biosynthesis pathways between RM_R and CK_R plants (Table 5). A total of 42 unique sequence that encode 13 enzymes were detected in the RNA-seq dataset. Comparative analysis showed that the expression level of most of the 42 unigenes is increased in RM_R plants, whereas only the FLS is decreased. Furthermore, five DEGs, including three key genes (*PAL, 4CL*, and *LAR*) and a cytochrome 450 unigene (*CYP98A3*) were obviously up-regulated in RM_R plants. In the present study, the levels of (+)-catechin and (−)-epicatechin in RM_R roots were significantly higher compared to those in CK_R plants, and the expression level of most unigenes related to the PAs biosynthesis were increased, particularly those encoding *LAR* (comp29969_c0 and comp29969_c1) (Table 5), which may have resulted in a markedly higher level of (+)-catechin in *F. dibotrys* roots. Interestingly, the absolute amount of (+)-catechin was significantly higher than that of (−)-epicatechin in both RM_R and CK_R plants (Table 1), suggest that the perennial buckwheat (RM_R and CK_R plants) may have different mechanisms for catechin and epicatechin biosynthesis. In addition, CYP450s are one of the largest gene families in plants and catalyzes most oxidation steps in secondary metabolism such as that involved in the biosynthesis of defense compounds, pigments, and antioxidants (Morant et al., 2003; Mizutani and Ohta, 2010). The present study determined that a cytochrome 450 unigene (*CYP98A3)* was significantly up-regulated in RM_R plants. The combination of various differentially expressed unigenes results in an increase in (+)-catechin and (−)-epicatechin levels in RM_R plants. Our data thus strongly support the correlation between the biosynthesis of active compounds and its related gene expression.

**Figure 7:**
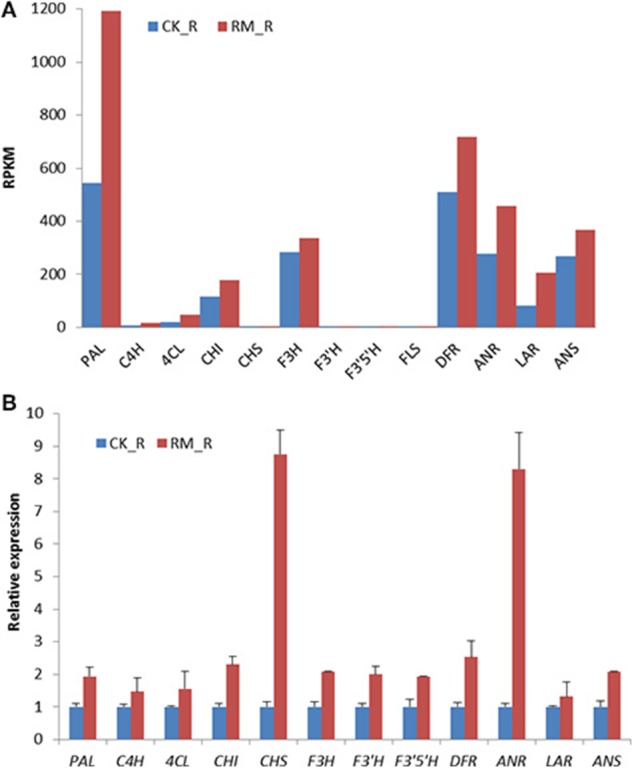
**Expression pattern of genes involved in flavonoid biosynthesis in RM_R and CK_R plants by RNA-seq and qRT-PCR analyses. (A)** Flavonoid biosynthesis-related genes detected by RNA-seq analysis. **(B)** Selected genes selected are confirmed by qRT-PCR analysis. The relative expression fold was calculated by using the 2^−ΔΔCt^ method. The Ct-values for unigenes in CK_R plants was arbitrarily chosen as the calibrator. Results represent the means ± standard deviation of triplicates.

To validate the RNA-seq data, the expression levels of 12 selected genes among the putative flavonoids biosynthesis-related genes in the roots of RM_R and CK_R plants were analyzed by qRT-PCR. The results were in general agreement with the observed changes in transcript abundance that was earlier determined by RNA-Seq analysis, and most of the genes involved in the flavonoid biosynthetic pathway were up-regulated in RM_R plants, which suggested that our transcriptome profiling data were highly reliable. *FLS* was detected at very low levels by qRT-PCR analysis and thus not represented in Figure 7. Discrepancies were only detected in four unigenes, whereas majority were up-regulated in RM_R plants, i.e., qRT-PCR analysis showed that the enzyme-encoding unigenes *CHS* and ANR were obviously up-regulated, whereas unigenes *PAL* and *LAR* showed moderately higher expression levels as compared to the RNA-Seq results. The differences in results were likely due to variations in platform employed in the analysis. The qRT-PCR primer sequences were designed for the 3′ ends of transcripts, which is highly stable, as compared to quantifying expression levels using the whole transcript with RNA-seq, which is typically not uniformly sequenced. In addition, we can infer that the *PAL, CHS, ANR, ANS*, and *LAR* genes were all involved in the flavonoid biosynthetic pathway and up-regulated in RM_R plants. However, the identification of unigenes playing more important roles in the PA accumulation process requires further validation.

### Discovery of candidate CYP450s and transcription factors with probable involvement in the PA biosynthesis in *F. dibotrys*

Cytochrome P450s (CYP450s) are reported to be nature's most versatile biological catalysts and form the biggest gene family in plants, accounting for more than 1% of the total gene annotations in individual plant species (Coon, 2005). These are generally involved in various biochemical pathways to produce secondary metabolites, which catalyze a wide variety of monooxygenation/hydroxylation reactions (Nelson et al., 2004). Gene co-expression analyses revealed that some *P450*s are coordinately expressed with key biosynthetic genes of various biological pathways. These genes are also the subject of analysis in various *de novo* transcriptome sequencing projects to unravel its novel functions (Gahlan et al., 2012; Gupta et al., 2013; Yang et al., 2013). The combination of high-throughput transcriptome sequencing and co-regulation analysis facilitates in the formulation of highly precise hypothesis on the function of uncharacterized biosynthetic genes (Gachon et al., 2005). An analysis of the expression pattern divergence of duplicated genes and/or gene families might also provide some insights into its metabolic pathways. Through Uniprot annotation, a total of 15 unigenes encoding CYP450 genes that were related to flavonoid biosynthesis were identified in *F. dibotrys* roots (Table 6). All the CYP450s involved in flavonoid metabolism in *F. dibotrys* were classified under 5 gene families, namely, *CYP73, CYP75, CYP76, CYP84*, and *CYP98* based on its diverse functions in flavonoid metabolism. Among all the *CYP* families classified, the maximum number of transcripts in *F. dibotrys* were those that belonged to that CYP73 family, with CYP73A transcripts as the predominant type. The function of the *CYP73A* unigenes that encode the enzyme, cinnamic acid 4-hydroxylase (*C4H*), is to catalyze *trans*-cinnamic acid to p-coumaric acid in the flavonoid biosynthetic pathway. The *CYP75A* gene, which encodes flavonoid 3′,5′-hydroxylase (*F3*′*5*′*H*) and belongs to the cytochrome P450 monooxygenase system, is the only enzyme that catalyzes the 5′-position hydroxylation of the B-ring. Wang's results showed that *CsF3*′*5*′*H* is a key controller of tri-hydroxyl flavan-3-ol synthesis in tea plants, which can effectively convert 4′-hydroxylated flavanone into 3′4′5′- and/or 3′4′-hydroxylated products, and is up-regulated in the flavonoid biosynthetic pathway of *Camellia sinensis* (Wang Y. S. et al., 2014). Recently, the roles of the *CYP82* and *CYP93* gene families were characterized in relation to flavonoid biosynthesis (Berim and Gang, 2013). These findings suggest that P450 genes involved in secondary metabolite biosynthesis are usually co-regulated with key genes in the biosynthetic pathway (Rastogi et al., 2014). The results also indicated that *CYP450s* play important roles in the PA biosynthetic pathway of *F. dibotrys*. However, the exact functions of these P450 genes in *F. dibotrys* requires further research.

**Table 6:** **Unigenes encoding cytochrome p450 involved in the flavonoid metabolism in *F. dibotrys* roots**.

**Gene_id**	**RPKM (CK_R)**	**RPKM (RM_R)**	**Gene Length**	**NR Description**	**Gene Name**
comp25500_c0	0.92	1.18	720	cinnamic acid 4-hydroxylase	CYP73A,C4H
comp26023_c0	2.5	2.41	1155	cinnamate-4-hydroxylase 2	CYP73A,C4H
comp37714_c0	33.9	28.52	1871	cinnamic acid 4-hydroxylase	CYP73A,C4H
comp38029_c0	282.21	246.94	1916	cinnamate 4-hydroxylase	CYP73A,C4H
comp38498_c0	7.29	13.32	552	cinnamate 4-hydroxylase	CYP73A,C4H
comp70348_c0	4.66	8.09	371	cinnamate 4-hydroxylase	CYP73A,C4H
comp7222_c0	6.29	14.61	254	cinnamate 4-hydroxylase	CYP73A,C4H
comp7222_c1	6.42	16.82	517	cinnamate 4-hydroxylase, partial	CYP73A,C4H
comp30892_c0	272.6	506.11	1916	cinnamate 4-hydroxylase	CYP73A,C4H
comp32658_c0	2.5	4.61	1732	PREDICTED: flavonoid 3′,5′ - hydroxylase 2	CYP75A F3′5′H
comp753300_c0	0	0.97	221	cytochrome P450	CYP76C
comp34829_c0	2.41	14.03	1797	ferulate 5-hydroxylase	CYP84A, F5H
comp40015_c0	23.77	31.51	1959	ferulate 5-hydroxylase	CYP84A, F5H
comp43448_c0	28.41	27.75	1583	coniferylalcohol 5-hydroxylase	CYP84A, F5H
comp35679_c0	31.55	83.17	1815	cytochrome P450	CYP98A3, C3′H

Transcription factors (TFs) are sequence-specific DNA-binding proteins that interact with the promoter regions of target genes to modulate its expression. In plants, several TFs play essential roles in the regulation of secondary metabolite biosynthesis and accumulation (Gantet and Memelink, 2002; Vom Endt et al., 2002; Zhou et al., 2014). As flavonoids are the main determinants of active compounds in *F. dibotrys*, it is essential to investigate the transcriptional regulation of genes involved in its biosynthesis, which can be further used in modulating the pathway and in developing flavonoid-enriched chemotypes. Anthocyanin pathway genes are regulated at the transcriptional level by three types of regulatory genes that encode R2R3 MYB, basic helix-loop-helix (bHLH), and WD40 proteins (Grotewold, 2005; Petroni and Tonelli, 2011). These regulators interact with each other to form a MBW complex that binds to promoters and induces the transcription of genes of the anthocyanin biosynthetic pathway. Several R2R3 MYB transcription factors have been identified in several model plants such as maize, *Antirrhinum*, petunia, and *Arabidopsis*. The increasing availability of plant genome sequences has allowed the identification and isolation of a large number of *MYB* genes involved in the regulation of flavonoid biosynthesis, from diverse non-model plants species such as grapevine (*Vitis vinifera*), strawberry (*Fragaria xananassa*), apple (*Malus domestica*), cauliflower (*Brassica oleraceavar botrytis*), potato (*Solanum tuberosum* L.), bayberry (*Myrica rubra*), mangosteen (*Garcinia mangostana* L.), pear (*Pyrus pyrifolia)*, and purple kale (*Brassica oleracea* var. *acephala f. tricolor*) (Hichri et al., 2011). Ma et al. (2009) cloned a putative *R2R3 MYB* protein named *FdMYB1* from *F. dibotrys*, and determined that the *FdMYB1* gene has the same classic characteristics as other MYBPs and thus is probably involved in flavonoid metabolism. We investigated the unique sequences that encode TFs in our *F. dibotrys* RNA-seq dataset in response to the irradiation exposure. A total of 452 unique sequences that encoded putative TFs were detected, which were then grouped into over 50 *TF* families, including 57 up-regulated and 19 down-regulated genes (Table 7, Additional file 8). The most abundant TFs was *EREBP* (58), followed by *bHLH* (48), *MYB/MYB*-like (46), *WRKY* (41), *TFIIP* (23), *GRAS* (21), *NFY*(19), *GATA* (18), *HSFF*(14), *bZIP* (13), *TCP* (11), and *R2R3-MYB* (9). Those that were annotated to have sequence-specific transcription factor activity but cannot be grouped together with any family were included in the “others” TF category by homology BLASTX analysis using the *Arabidopsis* transcription factors database (http://Arabidopsis.Med.Ohio-state.Edu/AtTFDB/) and plant transcription factor database (http://planttfdb.cbi.pku.edu.cn/) classification using the Itak software. A total of 10 *MYB* genes were up-regulated and 2 *MYB* genes were down-regulated. Other up-regulated transcription factors such as *R2R3-MYBP, bHLH, WRKY, TFIIP, NAC*, and *bZIP* have been reported to modulate genes involved in plant growth and plant response to biotic or abiotic stress. This information may be useful in further analysis of genes that are regulated by these TFs, particularly those associated with the flavonoid pathway.

**Table 7:** **Transcription factors identified in *F. dibotrys* roots using the RNA-seq dataset**.

**Putative TF family**	**No. of uniquence sequences**	**Up-regulated**	**Down-regulated**
MYB/MYB-like	46	10	2
R2R3-MYBP	9	2	1
bHLH	48	8	2
WRKY	41	6	3
EREBP	58	5	1
HSFF	14	4	0
GRAS	21	0	0
NFY	19	1	0
GATA	18	0	0
TFIIP	23	2	0
TCP	11	0	0
Trihelix	7	0	0
bZIP	13	1	1
AP2	7	0	0
ARF	5	1	0
GRF	1	0	0
NAC	7	2	0
MADS	4	1	0
C2H2	3	0	0
DOF	3	1	0
TGA	3	1	0
UNE	4	0	1
RF2B	4	0	0
PIFI	3	1	1
E2F1	2	0	0
KAN	2	0	0
ASG	2	0	0
HY5	2	0	0
BTF	4	0	0
GTE	3	0	0
VIP	2	0	0
HBP	2	1	0
BIM	2	0	0
Pur-alpha	2	0	0
SPATULA	2	0	0
RF2a	2	0	1
C3HL	1	0	0
C6	1	0	1
CBF	2	1	0
CCAAT	1	0	0
MEIS1	1	0	0
COL	1	0	1
CRT/DRE	1	0	0
UPBEAT	1	0	0
TFDP	1	0	0
MYC	1	0	0
TINY	1	0	0
YABBY	1	0	1
ZF	1	1	0
VRN	1	0	0
POSF	1	0	0
PAT	1	1	0
BEE	1	0	0
CPC	1	0	0
GLABRA	1	1	0
OTHERS	33	5	3
WD40	1	1	0
Total	452	57	19

Some members of TF families showed greater differential expression in RM_R vs. CK_R [*Q* < 0.005 and the absolute value of log_2_ (fold change)>1], suggesting that these played important roles in the induction of the flavonoid biosynthesis pathway. Among these TFs, 11 obviously differentially expressed unigenes in RM_R vs. CK_R were discovered to be related to flavonoid biosynthesis (Table 8), including 3 down-regulated unigenes (comp18844_c0, comp29626_c0, and comp41217_c0) and 8 up-regulated unigenes. These 8 up-regulated putative TFs were classified into MYBP(4), MYB-like(1), bHLH, and WD40(1) gene families, respectively. The expression pattern of these up-regulated TFs was in agreement with the above mentioned key genes that were related to the flavonoids in *RM_R* plants, which indicates that these TFs may have been conserved and played important functions in the regulation of flavonoid biosynthesis and accumulation. On the other hand, the down-regulated TFs, including MYB44, bHLH, and PIF1 gene families, were possibly responsible for down regulating or switching off sets of genes that were not related to the flavonoid biosynthetic pathway. The observed differences in the expression level of TF-encoding unigenes in RM_R and CK_R plants suggested that the metabolic regulation of *F. dibotrys* roots at harvest time was highly diverse. A systematic analysis of these TFs may help in better understanding the differential regulation of the flavonoid biosynthesis pathway in *F. dibotrys*. In addition, an in-depth analysis of the functions of these TF gene families that regulated flavonoid biosynthesis of *F. dibotrys* is warranted.

**Table 8:** **Differentially expressed unigenes encoding transcription factors involved in flavonoid metabolism in *F. dibotrys* roots**.

**Unique sequence ID**	**Length (bp)**	***E*-value**	**Gene family**	**RPKM_CK_R**	**RPKM_RM-R**	**log_2_ FoldChange**	***P*-value**	***Q*-value**
comp18844_c0	1150	2.04E-57	MYB44	56.7	6.89	3.0263	9.19E-11	1.16E-08
comp32832_c0	1440	2.98E-82	MYBP	9.54	42.9	−2.1846	1.04E-07	8.70E-06
comp41715_c0	1644	2.45E-123	MYB2	26.08	60.66	−1.204	8.78E-05	0.004255
comp21580_c0	1338	1.24E-83	MYBPA2	9.51	39.19	−2.0671	2.57E-06	0.000171
comp28772_c0	1444	6.04E-79	MYB-related	19.02	49.07	−1.3828	3.74E-05	0.001976
comp29046_c0	1508	4.05E-91	MYB/TT2	54.47	152.92	−1.5052	1.67E-15	3.55E-13
comp29626_c0	1953	2.44E-63	bHLH	107.83	48.33	1.1409	1.49E-10	1.85E-08
comp35865_c0	988	5.39E-58	bHLH	106.54	261.19	−1.307	4.27E-13	6.92E-11
comp32216_c0	1228	1.05E-30	bHLH	28.79	252.32	−3.137	5.77E-42	3.60E-39
comp41217_c0	2651	7.17E-56	PIF1	16.51	2.26	2.8633	3.65E-07	2.79E-05
comp43264_c1	2348	0	WD40	15.9	47.59	−1.7927	4.08E-07	3.10E-05

## Discussion

Flavonoids are a group of bioactive substances that have special health care and therapeutic functions, as well as play an important role in the production of functional foods and in the medical industry. Several plant culture practices aiming to increase rhizome yield and flavonoid content in *F. dibotrys* have been studied. However, molecular biology techniques have been scarcely used in the improvement of flavonoid accumulation in *F. dibotrys*, mostly due to the absence of available sequence information. Only 30 sequences of *F. dibotrys* have been submitted to the National Center for Biotechnology Information (NCBI) database. Due to the low number of NCBI sequences on *F. dibotrys*, it is difficult to isolate functional genes that influence its quality and agronomic traits.

Ultra-high-throughput mRNA sequencing technology has been previously used to analyze gene expression profiles of closely related species or different tissues in insects and plants (Swarbreck et al., 2011; Rastogi et al., 2014). Particularly in medicinal plants, the number of sequenced transcriptomes has skyrocketed in the last few years and continues to increase at a rapid pace. One of the most important goals of transcriptome sequencing is to explore specialized pathways of metabolite biosynthesis in non-model medicinal plants (Xiao et al., 2013). The roots of *F. dibotrys* have been used as medicinal material, therefore, in the present study, two samples from RM_R (irradiation-induced) and CK_R (the control) roots with significantly different active compounds (Table 1) were harvested at the same developmental stage, subjected to transcriptome sequencing, and then compared. We obtained about 4.14 Gbp of clean reads and generated a total of 53,540 unigenes by *de novo* assembly. To improve the accuracy of *de novo* assembly and to increase the effectiveness of gene annotation, we collected *F. dibotrys* tissues for RNA preparation at the usual harvest time, when the levels of its active compounds are higher than that at other time points. Additionally, a paired-end library sequencing strategy was applied not only to increase sequencing depth, but also to improve the efficiency of *de novo* assembly, and all seven public databases were selected for gene annotation comparisons to acquire comprehensive functional information. A total of 29,901(55.84%) unigenes were annotated based on BLAST searches against the public databases, and the annotated unigenes were used to study the secondary metabolic pathways. The remaining 23639 unigenes (44.16% of all assembled unigenes) did not generate significant homology to existing genes. The absence of homology could be caused by two factors. One factor is that a large proportion (31.3%) of unigenes was shorter than 500 bp, some of which were too short to allow statistically meaningful matches. The other is that for some unigenes, the absence of homologous sequences in the public databases may be indicative of its specific roles in *F. dibotrys*.

The main bioactive constituents of *F. dibotrys* were the end-products of flavonoid secondary metabolism. Irradiation can induce the biosynthesis of secondary metabolites and stimulate the accumulation of these compounds in plants (Zhao et al., 1998). Our previous studies illustrated that irradiation led to the accumulation of (−)-epicatechin in *F. dibotrys* rhizomes (Jia and Li, 2008). The main active flavonoid ingredients in *F. dibotrys* roots are the PAs, which result from the polymerization of flavan-3-ol units. The biosynthetic pathways that lead to the production of flavan-3-ols [(+)-catechin and (−)-epicatechin], which are the building blocks of PAs, have been extensively studied in plant models such as maize (*Zea mays*) and *Arabidopsis*. However, an assay for the PA biosynthetic pathway in *F. dibotrys* is currently not available. The annotation processes for DEGs provide a valuable resource in identifying specific genes and signal transduction pathways that are related to PA biosynthesis in *F. dibotrys*.

Detailed functional information is essential in better understanding the overall expression profile of *F. dibotrys*. The present study identified the levels of active compounds in the two samples (RM_R and CK_R), which were significantly higher in *RM_R* plants compared to those in *CK_R* plants (Table 1). The purpose of this research was to clarify the molecular mechanism of PAs (especially EC and C) biosynthesis, Here, we focused on identifying and comparing unigenes that may encode enzymes that participate in the biosynthetic pathways of PAs between *RM_R* and *CK_R*, and the assembled sequence information of these unigenes involved in the PAs biosynthetic pathway can be seen in the additional file 9. We further investigated these genes using the RNA-Seq analysis to obtain more information on its transcription profile. All of the reported genes related to the flavonoid biosynthesis such as *PAL (Comp40695_c0*, 99% identity to GenBank Accession number HM628904.1; *Comp40849_c0*, 80% identity to GenBank Acc. No. HM149783.1; *Comp41942_c0*, 92% identity GenBank Acc. No.HM149783.1; *Comp42986_c0*, 99% identity to GenBank Acc. No.HM149783.1), *C4H (Comp37714_c0*, 97% identity to GenBank Acc. No.HQ828144.1; *Comp38029_c0*, 91% identity to GenBank Acc. No.AM887638.1; *Comp38498_c0*, 99% identity to GenBank Acc. No.JQ946537.1; *Comp7222_c1*, 81% identity to GenBank Acc. No.GQ844863.3; *Comp70348_c0*, 78% identity to GenBank Acc. No.AM284167.1; *Comp30190_c0*, 98% identity to GenBank Acc. No.JX500528.1), *4CL (Comp43080_c2*, 97% identity to GenBank Acc. No. HQ840696.1; *Comp37790_c2*: 92% identity to GenBank Acc. No. HM149785.1), *CHS (Comp40750_c0*, 100% identity to GenBank Acc. No.GU169470.1; *Comp19447_c0*, 71% identity to GenBank Acc. No.FJ197128.1; *Comp2823_c0*, 90% identity to GenBank Acc. No.EU650636.1), *CHI (Comp35337_c0*, 99% identity to GenBank Acc. No.KF831243.1), *F3H (Comp44775_c0*, 98% identity GenBank Acc. No.HQ003252.1; *Comp30439_c0*, 97% identity to GenBank Acc. No.FJ456858.1), *F3*′*H (Comp38797_c0*, 98% identity to GenBank Acc. No.HQ003253.1), *FLS (Comp31227_c0*, 82% identity to GenBank Acc. No.HM357805.1), *DFR (Comp29690_c0*, 99% identity to GenBank Acc. No.EF522146.1), *ANR (Comp44789_c0*, 96% identity to GenBank Acc. No.KC404848.1), *ANS (Comp30968_c0*, 98% identity to GenBank Acc. No.KF938554.1), and *LAR (Comp35199_c0*, 98% identity to GenBank Acc. No. JN793953.1) were identified in the RNA-seq data, suggesting that these were all expressed in *F. dibotrys* roots, and some of these genes apparently formed multi-gene families. These findings implied that the buckwheat genome, similar to various other higher plants, underwent at least one round of genome duplication during evolution, whereas the functions of these genes require further research.

To investigate the molecular mechanism responsible for the variable levels of its active medicinal compounds, further analysis was conducted. Although the amount of PAs [(−)-epicatechin and catechin] in RM_R plants was all significantly higher than that of CK_R, some of the up-regulated unigenes of RM_R vs. CK_R were likely to be directly or indirectly involved in PA biosynthesis. The synthesis pathways of PAs and anthocyanins share common steps that lead to flavan-3,4-diols (such as leucoanthocyanidin), which can be converted to catechin (2,3-trans-flavan-3-ol) by LAR (Tanner et al., 2003) or to anthocyanidin by ANS (Saito et al., 1999; Abrahams et al., 2003). Anthocyanidin then either serves as the substrate for the synthesis of epicatechin (2,3-cis-flavan-3-ol) by ANR (Xie et al., 2003) or can otherwise be converted to anthocyanin by glycosylation (Schijlen et al., 2004). Both catechin and epicatechin act as the initiators of PA polymerization, with intermediates derived from leucoanthocyanidin, catechin, or epicatechin added sequentially as extension units. We identified DEGs that encode for enzymes involved in the PA pathway. However, a total of 501 differentially expressed unigenes in RM_R and CK_R were identified. The down-regulated and up-regulated unigenes of RM_R vs. CK_R were enriched in the biosynthesis of other secondary metabolites and metabolic pathways, respectively. The gene expression profiles of the roots of *F. dibotrys* were obtained, and key putative genes such as *DRF, ANR*, and *LAR* that may be involved in the biosynthesis or regulation of vital medicinal metabolites were identified. In the present study, unigenes *DFR, LAR, ANR*, and *ANS* were all up-regulated, which may result in an increase in the level of PAs [(−)-epicatechins and catechins]. *DFR* is the first committed enzyme of the flavonoid biosynthetic pathway that generates common anthocyanins, and is a rate-limiting enzyme in the biosynthesis of anthocyanins and condensed tannins, and catalyzes the reduction of dihydroflavonols to leucoanthocyanins (Huang et al., 2012). Its catalytic reaction is an important regulatory step. Induction of *DFR* activity has been linked to an increase in the accumulation of condensed tannins (Peters and Constabel, 2002; Cheng et al., 2013). The enzyme activity of *ANR* has been detected in various plants such as grape (*V. vinifera*), soybean (*Glycine max*), tea (*C. sinensis*), and legume (*Medicago truncatula*) (Bogs et al., 2005; Pang et al., 2007, 2013; Kovinich et al., 2012). The functionality of *LAR* has been reported in several plant species and its activity is correlated with PA accumulation (Pang et al., 2007, 2013; Gagné et al., 2009). Our data strongly support the correlation between metabolic pathways and its related gene expressions.

Most plant medicinal components are secondary metabolites whose biosynthesis largely depends on cellular responses to external signals. Recent studies involving transcriptome sequencing of medicinal plants have shown that not only the enzyme-encoding genes but also regulatory genes contribute to the production of active medical components (Sangwan et al., 2013). In the present study, Some cytochrome P450 genes were discovered in the transcriptome of *F. dibotrys* roots (Table 6), and the expression level of most unigenes encoding cypt450 genes were slightly or more increased in RM_R plants. In particular, the unigenes comp7222_c0, comp7222_c1, comp30892_c0, comp34829_c0, and comp35679_c0 were expressed at higher levels in RM_R plants than in CK_R plants [*Q* < 0.005 and the absolute value of log_2_ (foldchange)>1]. The differential expression patterns of these cytochrome P450 genes in RM_R and CK_R roots were indicative of its contribution to the biosynthesis and accumulation of flavonoid secondary compounds. Identifying the molecular mechanisms underlying differences in the levels of the main medicinal components between *RM_R* and *CK_R* requires further experiments, whereas the identified unigenes involved in these biosynthetic pathways may be highly useful in future investigations. Modulation of gene expression is a critical component of cellular responses. Some TFs directly regulate gene transcription and the expression of metabolites biosynthetic enzymes (Galis et al., 2006; Bomal et al., 2008). Therefore, the identification and isolation of TFs is important, which in turn indicates that further verification of its differential expression profiles are necessary. In our comparison of the unigenes encoding TFs from RM_R and CK_R, 11 were differentially expressed and related to flavonoid biosynthesis (Table 8), including 3 unigenes that were down-regulated, and 8 unigenes that were up-regulated. These 8 up-regulated putative TFs were classified into the MYBP(4), MYB-like(1), bHLH, and WD40(1) gene families, respectively. The flavonoid pathway appears to be mainly regulated at the transcriptional level (Brenda, 2001). Several TFs have been isolated in a diverse group of plants (Davies and Schwinn, 2003; Koes et al., 2005; Allan et al., 2008; Palapol et al., 2009; Niu et al., 2010) that control its transcription. In particular, interacting R2R3-MYB and bHLH type TFs form a complex with WD40 proteins (termed the MBW complex) that activate anthocyanin and proanthocyanidin biosynthetic genes. The MBW complex usually regulates groups of flavonoid biosynthetic genes that vary among species (Davies and Schwinn, 2003). This regulation is via specific binding to motifs in the promoters of the pathway genes (Hernandez et al., 2004; Hartmann et al., 2005; Dare et al., 2008). In the present study, most putative TFs were differentially expressed, which indicates that these TFs played important roles in irradiation-mediated bioactive compound biosynthesis and regulation in *F. dibotrys*. However, whether fine regulation of the flavonoid biosynthetic pathway genes in irradiation-induced roots involves a combinatorial action(s) of different transcription factors, including basic helix-loop-helix (bHLH) and WD40 factors could not be determined. A more in-depth analysis is needed to determine the functions of these gene families that have been associated with molecular and cellular changes occurring in *F. dibotrys* roots in response to irradiation.

Finally, we selected twelve 12 enzymes to validate our gene annotations. qRT-PCR analysis showed consistent expression patterns. We are confident that our transcriptome database is a valuable addition to the publicly available buckwheat genomic information. Our work generated a large set of cDNA sequences that represent the flavonoid biosynthesis pathways of known plant genes and potential new genes in the genus *Fagopyrum*. This study provides an overall picture of the variation in the expression of biosynthetic enzyme genes between the two samples.

To sum up, RNA-seq technology is a fast, efficient, and cost-effective way of characterizing the transcriptome. It is especially suitable for gene expression profiling in non-model organisms that lack genomic sequence. We obtained a total of 53,540 unigenes using the Illumina HiSeq2000 sequencing platform from *F. dibotrys* roots collected at the harvest time, of which 29,901 (55.84%) could be annotated based on the BLAST searches against public databases, and 501 unique sequences were differentially expressed between the samples of RM_R and CK_R. Twelve unigenes encoding TFs (e.g., MYB, bHLH, and WD40) were differentially expressed between the RM_R and CK_R samples. In addition, some candidate cytochrome P450s (*p450s*) that could be involved in flavonoid accumulation of *F. dibotrys* were selected from the RNA-seq data. Importantly, qPCR detection showed that the expression patterns of 12 of the key candidate genes that were related to the flavonoid biosynthetic pathway were in agreement with the RNA-seq data. Genes that encode key enzymes with different expression patterns between RM_and CK_R were discovered, which was suggestive of the complexity of flavonoid biosynthesis in *F. dibotrys*. Several candidate *P450* genes, which were mined from the transcriptome data, provided the presumptive enzymes for the remaining steps in the flavonoid biosynthetic pathways. Further analysis of TFs could aid us in understanding the regulatory patterns in *F. dibotrys*. In summary, irradiation increased the expression level of some key genes in PA biosynthesis, which in turn resulted in elevated rates of PA accumulation. This comprehensive investigation sheds light on the molecular mechanisms underlying radiation-mediated bioactive compound biosynthesis and regulation in *F. dibotrys*. The transcriptome data from the present study yielded new insights on the process of PA accumulation in *F. dibotrys* root.

## Author contributions

CC participated in the design of the study, contributed to the tissue sample collection, RNA extraction and cDNA library construction; analyzed the data, discussed the results and drafted the manuscript. This work was conducted in the laboratory of AL who contributed to the evaluation, discussion of the results and revision of the manuscript. The authors declare no conflict of interests and thanks Ms. Hongmei Luo (IMPLAD) for her help.

### Conflict of interest statement

The authors declare that the research was conducted in the absence of any commercial or financial relationships that could be construed as a potential conflict of interest.
