# Supplementary material for: Transcriptome Analysis of Differentially Expressed Genes Involved in Proanthocyanidin Accumulation in the Rhizomes of Fagopyrum dibotrys and an Irradiation-Induced Mutant
Source: Front Physiol. 2016 Mar 18;7:100. doi: 10.3389/fphys.2016.00100 (PMC4796566; doi:10.3389/fphys.2016.00100)
Supplement: Supplementary file 2 [file Presentation2.ZIP › Supplementary Material/Additional file 9. Sequence information of PAs biosynthetic genes in F. dibotrys roots.docx]

***PAL***

CGGGGAAGGAAAATATTAATAACCCTCTCGCAGTATTTTCCTAACACAAAACCCAAAACCAACAAAAAAATAACAACAAGCTCGTGAATTCGTGAACATATCTATCATCTCTTGATCGAAATGGAGGTCTCAAACGGACATCGCAACGGAAACGGAATCGTAGCCAACGGGCTTTGCTTGAAGAAGGAGTTGTCGGGAACTGTGCAGGATCCGTTGGGGTGGTTGAAGGCGGCGGAAGGGATGAAAGGGAGTCATCTGGAGGAAGTTAAGAAGATGGTGGAGGAGTTTAGGAATCCGGTGGTGAAGCTCGCCGGAAAGACTCTTAGCATTGCGCAGGTGGCTGCGATTGCTGCTTCCGGTGAGGGCGGTGTGATTGTCGAGCTGGATGAGGAGGCTAGACCCGGGGTTAAGGCTAGTAGTGACTGGGTTATGGATAGCATGAACAAGGGAACTGATAGCTATGGAGTCACCACTGGATTTGGAGCTACCTCTCATCGTAGGACTAAGGAAGGTGGTGCTCTTCAGAAGGAGCTCATCAGATTTCTGAACGCCGGAGTATTCGGCAACGGAGTGGAATCATGCCACACGCTCCCGGCGACCACCACCAGAGCTGCTATGCTTGTACGGATCAACACTCTTCTCCAGGGATACTCTGGTATCAGATTCGAGATCCTCGAAGCTATTGCCAAGTTCCTCAACCACAACATCACTCCTTGCCTCCCACTCCGTGGAACCATCACTGCTTCTGGTGACCTTGTTCCTCTGTCCTACATCGCTGGTCTTCTCACTGGACGTCCTAACTCTGTCGCAGTTGGTCCTGACGGCCGCTGTATGTCTCCCTCTGAGGCCTTCGAAGTTGCTGGAATTGATTCCGGATTCTTTGAGCTACAGCCTAAGGAAGGTCTTGCTATGGTTAATGGAACTGCTGTTGGATCTGGTCTTGCCTCCATTGTGCTATTCCAGGCGAATCTCCTTGCTGTTCTCTCTGAGGTTATCTCTGCTCTCTTCGCTGAGGTCATGAATGGAAAGCCTGAGTTCACTGACCATCTGACTCACAAGCTGAAGCATCACCCCGGCCAGATCGAGGCAGCTGCTATAATGGAGCACATACTCGACGGATCTGGCTACGTCAAGCACGCTGAGAAGCTTCATGAACTCGATCCTCTCCAGAAGCCAAAACAGGATCGTTATGCGCTCCGAACTTCTCCTCAGTGGCTCGGCCCCCAAATCGAAGTGATCCGTGCGGCTACAAAGATGATTGAGAGGGAAATCAACTCCGTCAACGACAATCCGCTGATTGATGTTGCTAGAAGCAAGGCTTTGCACGGAGGAAACTTCCAAGGGACACCAATCGGTGTTTCCATGGACAACACCAGGCTTGCCCTAGCCTCGATCGGAAAGCTGTTGTTCGCTCAATTCTCTGAGCTCGTCAACGATTTCTACAACAACGGACTGCCTTCGAATCTCTCCGGTGGGAGAAACCCTAGCTTGGACTATGGATTCAAGGGAGCCGAGATCGCTATGGCTTCATACTGCTCTGAGCTCCAATTTCTGGCGGATCCAGTGACTAACCACGTCCAGAGCGCCGAGCAGCACAACCAGGACGTGAATTCCTTGGGACTGATCTCATCAAGAAAGACCGCAGAGGCAGTTGAGATTCTCCAGCTCATGTCTTCCACATTCCTTGTTGCTCTCTGCCAGGCAATCGATCTGAGGCATTTGGAGGAGATCCTTAGGAACACTGTGAAGAACACAATCAGCCAAGTGGCGAAGAGAATCCTGACAGTAGGAGTGAACGGCGAGCTCCATCCAAGCAGGTTCTGCGAGAAGGAACTCCTTAGGGTTGTGGATCGCGAACATGTCTTCGCCTACATTGACGATCCTTGCAGCGCTAACTACGTCCTAATGCAGAAGCTCAGGCAGGTGCTGGTGGATCACGCTTTGGCAAACGGAGAAAGGGAGAAAAACAACTCAACTTCAATCTTCCAGAAAATCGGAACATTCGAGGAGGAACTCAAGACTGTTTTGCCAAAGGAGGTTGAATTAGCAAGAACTCTATACGACAACGGAGCTTCTGGTGTGGAGAACAGGATCAAGGAATGCAGATCTTACCCGCTGTACAAGTTCATCAGGGAGGAGCTCGGTACGAGCCTGTTGAGTGGTGAGAATGTTCGATCACCTGGAGAGGATTTCGACAAGGTGTTCACTGCCCTCACTGGAGGATTGGTGATTGATCCTTTGCTTGAGTGCTTGAAGGAGTGGAATGGTGCTCCTCTGCCTATCTGCTAGGTCTGCTTTCATCTGTTTTTACATGTTGCTTTCATGAACATCAATGGGTTTAATATTTTGGCTTTTTTTTTTTATTCTGTTGTTTAGTTTTTTCTTCTTCTTATATTTAAGTTTACAGTGTATGTAAGGGAAAGCAGTAATGTTCTTGAATCTGAATTTGTTCTTAAAGAATGAAAAGCAGCTATATTCGATTATATATATATATACCTTTTGCCTTTGTTTTGTTTTGTTTTTGTCAAACTTCTAAG

***C4H***

GTAGCCATTGCTGAGATAATAAAGGTGAACATCTCTCTGTGTTTGGATACTGTGAGACAACTGATTTTCCTTCACTATTTCTTATTCAAGCAATTTTAGGAACCATCTTAATTATGCAACTTAAATATCTCTACGCATGAACTTAAGCATAAATTTGTACAAAATCTCTTCTGCAGGGTCTGGCTTATAAGATTCTTAAAAACACAATTTTTAACAATACTCATTAGTTAAGCTGATTTTTTGGTTTTTTGTATTTGTATACTAGACAAAGACTCCTTCCATGCTTGGATCGAGTTAATTGTCCTCATTGTAAGTAGCTCTCTTGAAAACTCTTTCCATGCCATATGATTGTTTACACTCTGCATTTAGGTTAACTGTGATGAATAAGTTTGTGTAGTTTTACACGTAATTTAACCTTTTCTTGATAGATGGATGTTCTTTCCCTGTTAA

***4CL***

GACTACGAATTCGTTGCCATGTTTGATGCAGATTTCCAGCCCACATCTGATTTCCTAATGAGAACTGTCATCCATTTCAAGGACAATGAAGAAGTGGGTCTTGTTCAAGCTAGGTGGTCCTTTGTAAACAAGGACGAGAACTTGCTAACAAGGCTACAAAACATTAACTTGGCCTTCCATTTTGAGGTGGAGCAACAAGTGAATGGCATCTTCCTTAATTTCTTTGGGTTCAATGGCACTGCTGGCATCTGGAGAATTAAGGCATTGGAGGAGTCGGGTGGTTGGCTTGAGAGGACCACAGTTGAGGATATGGACATTGCAGTTCGTGCCCATCTACACGGATGGAAGTTTGTCTTCCTTAATGACGTTGAGTGTGAATGTGAGTTGCCAGAATCATACGAAGCTTATAGGAAACAACAGCACAGATGGCATTCTGGGCCTATGCAGTTATTCCGCCTTTGCTTACCAGAAATATTCAAATCAAAGATTGGTGTTTGGAAGAAGGCAAATCTGATATTCCTCTTCTTCCTCCTGAGAAAATTGATCCTGCCATTCTATTCCTTCACACTATTCTGTATAATCTTACCAATGACAATGTTCGTGCCAGAAGCAGAGCTCCCAGCTTGGGTTGTCTGTTACATTCCAGCAACCATGTCATTCCTCAACATCTTGCCAGCTCCAAAGTCCTTCCCTTTTATTGTCCCTTACCTTCTATTTGAGAACACAATGTCCGTGACCAAGTTCAATGCAATGATTTCGGGTCTTTTCCAACTCGGGAGCGCATATGAGTGGGTTGTGACCAAGAAATCAGGTCGGTCTTCTGAGGGCGACCTCGTCTCTCTCGCTACTTCCGAGAAAGAGGCAGCCCTTCGTCATAGGAGCTCCTCTGAATCCGATCTAGTTGAGGTAGAGGAAAAGGGTTTGAAGAAGATGAATAAGAAGAAAAAGAAACACAACCGGATATACATGAAAGAATTGTCTCTTGCTTTTCTTCTTCTCACAGCTGCAGCCAGAAGCTTGCTTTCTGCTCAGGGTATTCACTTCTACTTTCTTCTCTTCCAAGGAATCTCCTTCTTACTTGTTGGTCTAGACTTGATCGGCGAACAGATTAGTTGAGTAAAAATGAAAAACGGTAGCCGGGTTTGTAGGAAAAATACGCATATATAGTAGTAAGAACAATGCAGCGCACGCGATTAATGCGTTTGCATCCTCTGCCTCTCGGTGCTCAAGTGTCAACTGAGCCGAGTTATGTCGGGATCAAGATTTGAATCCGAAGCGAGAGAAGAGAGGGAGGTCATCTGAACTCGATGAACTCCCATGGTGGATAGGGAAGAAAGTTTTTTAGGCAATGGTTTTCCCTTTGTTTTATGTTGATTTCTGTATGTAATTTGATGGGTTTTGATTCTTTTTACTTCCCCTGTTTCTGGGTTTATTCTTTTTGTAAGAAAGAAGAGAAAATTGCTGTCTAGAGATGAATAAGTTAAGTTGTTACTTCACATTTGGTTAAAGCAGAGTTCTTGGATGGAATAAAATTGGCATGATGAGCCATTGAGTTTGCAGGACAAAAACTTGTGTTATCCGATTGGAAATGCAAATACAGAAAGAAATGGTGATGTTCAGTGGGCTGAAATGAAATGGCCATACACAAAGGGAGACAAATGCATGTGAAGACTTGGTAAACACAGAAAGTGGGCAAGGAGATAGGAATCTCTCTGTAGAGTTGTTAAGTGTGTAATCTGGCTGAGGAAATTTATGTGTATGGGGCCATCTTTTGGATCTTTTTGAGGGTTGTCCAACATTTCACATGGCTCACTCTTTTAATCTTTAAAATATTGGAAACGACTCTGTAATTTGCTCTTTTACTCCATCTTTTAGTCTGTAATTTCCATATACATGGTTTCATCCAACTAAGACAGATTTGACTGGGGTC

***CHS***

GAAGGTTGGTAGCTGGCACGTACTTAGCTAGCAGCCGAGCAGAGTGAAAAGTAAAGTCCAAGTGCCTAACACCTCACGTGATCACTCAGAAAGACAGAAACAACATCCATCTACCCGTCCAGTCCTCACTCCTTAGAGAATCACCTACCCACCACCCTTCGTTCATAACTCCGTATAGATATATATATACACACCCAACCCAAGAGAGAGATCAAACAACAAAACCACACAATTAATTAACCATCTCCTCCCTTCCAACAAATTTTAAACTTGGATCGAAATGGCACCGACGGTCCAGGAGATCAGGAAGGCTCAGAGGGCCGAGGGTCCGGCGACCGTGCTGGCAATCGGGACGGCGACGCCTCCCAACTGCGTCTACCAGGCCGACTACCCCGACTACTACTTCAGGGTCACCAACAGCGACCACATGACCGACTTGAAGGAGAAATTCAGACGCATGTGCGACAAATCACAGATTGAGAAGCGTTACATGTACCTAACCGAGGACATCCTCAAGGAACACCCGAACATGTGCGAGTACATGGCGCCGTCTCTAGACTCCCGCCAGGACATGGTCGTCACCGAGGTTCCCAAGCTCGGCAAAGAGGCTGCCCAGAAGGCCATCAAGGAGTGGGGCCAGCCCAAGTCCAAGATCACCCACGTCATCGTCTGCACCACCTCCGGCGTCGACATGCCCGGAGCGGACTACCAGCTCACCAAGCTCCTCGGCCTCCGCCCTTCCGTCAAGCGTTTCATGATGTACCAGCAGGGTTGCTTCGCCGGAGGCACCGTCTTGCGTATGGCCAAGGACCTCGCCGAGAACAACAGGGGGGCCCGTGTCCTCGTCGTCTGCTCCGAGATCACCGCCGTCTGCTTCCGTGGACCCACCGACACTCACTTGGATTCCATGGTGGGCCAGGCCCTCTTCGGGGACGGAGCCGGAGCGGTTATAGTCGGAGCGGACCCCGACCTCTCCATCGAGAAGCCCATCTTCGAGCTCGTTTGGACTTCCCAGACCATCCTCCCAGACTCCGAGGGTGCCATCGACGGTCACTTGCGCGAGGTCGGACTCACCTTCCACCTTCTCAAGGATGTCCCCGGCCTCATCTCGAAGAACATCGAAAAGAGTCTTACCGAGGCCTTCTCCCCTCTCAACATCGCCGACTGGAACTCCCTCTTCTGGATCGCCCACCCCGGGGGCCCCGCAATCCTCGACCAGGTCGAGGCCAAGCTCGGACTCAAGGAGGAGAAGCTCAAGGCCACCAGGCAGGTCTTGAATGACTATGGTAACATGTCCAGTGCCTGTGTTTTGTTCATCTTGGATGAGATGAGGAAGAAGTCCCTCGAGAACGGACACGCTACCACCGGAGAGGGTTTGGATTGGGGTGTCTTGTTTGGCTTCGGACCCGGACTTACCGTCGAGACCGTCGTCCTACACAGTGTCCCCACCACCACCCTAGCCAACTGATCCATTTAAGCTTGCTTCCTCCCTCGGCGCCCTTACATTTTCAGTACGTCTTAATTAAGAAGCAATGCCATCAACTCCCACGATAGACCATCGATTTTTTTTTTGGTTCACATATTCACATTATATGTTTCGCTTCTTTGTCTTATCTTATAAGTAAGGTTTCTTTTCTATTTTATTTCAGCCTAGTGTGATGAATATGAAAGTTGAATTATTGTCAAAGCTCTAATAAAATACTTTCACTCAATCTTCCTCCTTAGTCGATCACATGACCCGG

***CHI***

GTCCAATTTAAATGAAAGTTCAACGACGACTGAAAGCACAAGTTGATGTGCATCGCCAACCCGACGATCCAATAAAAAACTATCTACCCCAATTTTGAATTTTGTTGGTCCATATAAAGTCCTCCAACGCTCCAGGATGGTTGCTTATGAGTTACATTTGGTGTTCCATGTCTCATTAATTCGACAAACAAGAGGCCATTGACACTGCACGGTAAGTGATTCCTTCAGATTCACCGTTCAAGCACAACAAGTGTTGGCTGAGACAATCACTAAAATTGAGGAATTTAAGTTTAACTGCATAACTACTGATGTGTTATTCATAGTACACATGAAGCAAAAACACAATCAGAAAATGCCAAATCATATTGAATCATTCTTTGATCTCCACACCATTCTCTTTGGTTACAGCTTCAGCTTCTCCATTTGCAACTTTGGTTTCAGCTTCAGCTTCTCCATGTGCAACTTTGGCAGGATTCAGCAATTCATGGAGTCTAACAGCCAAGCTCTCCTTGGCTGCTGGCGAAACGCCATTCTTACCGATTATAGATTCTAGAACAGATTGCGACAAAGGCCCGTTTTCAATCACAGCAACATCAGCTTCTGGTATGGCATCATGCTTCCCGAATGCAATCCTTAATGATTTTGGGGCGCATTGCTTGAACAAAATAGAGGTTCCAGGGGGGAAGTTCTGTTCTTTGAAGATCTCTGTGAATTTTTCTATAGCTTTCTCTTCAGCTTCAGAGTAGATTCCAATTGCTTTCCATATAGCTACGCAGTTCTCTGATACCTTTTCAGAGTACTGTGCCCCAGTTAAGGGCTTCAACATGGTGATCTGTATGAATTTTTCGAATTGACCAGTGACAACATCTCTGAAAAACTCAACAGATTCAGTCAACTCGGTGGCGGATTTCCCCTTCCACTTGTCAGCGAGTGACGCCACGGCGGTTTCCTCAAAGTAAATTCCGATCGCCGTGAAGGATATGAATGTACCATTGATCGTGAGCCCCCTAACTCCTGCACCGCCGAGAAAGAAAGACTTATCGGTGGCGGGAGGGCGGACGGAAGGAGGAAAGACGAAATCCTCGATGGCTATGGAGGATACGGTGATTGATGAAGCCATTGTCGGTAGTGAGTGTAGTGTCTGGGGTGGTTGAGGAGAGCACGAGGATGAAGAGAAGGGGACTGGCAGATTTCCACGGTTTTCATTCAATGGAATTTATAGATAGAGAAGATTTGAAGAGAGAGAAAGTGGGATAGAGATCGGGGTAGTTGTGACTTGTGAG

***F3H***

TTAACCTTGGAGGTTTTCCCTTCTTTGTTGTGGCATTAGTTCACACTTCACACATTGTTTGTTGAGAAAGAAGAAGTTTTACATACAGCGTTAGACTCTCCCTCTTCTTCTTTTTTTCATTTCATCTTTGACCCTTGAACTTCGTAAACTATTGAGCTAAAACTATGTATATATCAGTGACGACTGATGACCCTAAATTCAAATATCACCAGTGAACAATACAGGTCCAAGAGATTTATTCAACTTCCACCATCCAAACTTCCAGTTACAGAACACATTTCAACAGACGTTTAAATAAAAGAAACAGAGCAGAACAGAGCATAAGAGAACATAATATCATCTTCTTTAGCTAATCATAGAAAACCCTAATGTGTTCAAAAAGGCGTTTATCCAATGAGTAAAGTAGTTTCAAGCAAGGATCTCTTCAATAGGCTTAGGTTCCACCTTTGCCTTGTCGAGATTCTGCTGAGCTTGCTCCTTGGCTAGCTTCTTCAGCCTTGCAAGCTCAAGATCCTTACCCATCTTTCTCCTGTACATCTCAGCAAAGGTGATTGGTGCCTCTAGAATTGGCGTCTCGCCTTCCCTCACTGCTAGCGGGTAAACGGTTGCATCTGGGGCCGGGTTTTGGAATGTGGCGATTGAAAGACGGCTGAAGTTTGAGTTCACCACAGCTTGGTGATCAGCATTCTTGAACCTTCCATTGCTCAAGTAATGACCATGGTCACCAAGGTTAACAACAAAGGCACCCTCAACAGGTTGAACAGTGATCCAGGTTTTGCCATCATCTCTAGTAGCCTGGAGTCCACCAACTTGGTCTTGAAGCAAGAGGGTGATGGTACCCGGGTCAGTGTGACGCTTGAGACCAAGGGTGAGGTCCGGCTGGGGGCATTTCGGGTAGTAGTTTACAACCACCTTTTGGTCCATGTCGACACAAGCCTTGGTCAAGGCCTCGGTCTCGAGTCCCATTGCTTCTGAGAGAACTCCAAGAAGCTTGCAAGCTAGACCCATGAGCTGCTCGCTGTAGTGCTCGGTTACCTTGACCCAGCCCTCGGGCTTGTCGGGCCACCTTGAGTAGTCCCTGGCCTTGAGTGGGTATGAGAAGTATGTCACTATCTCACGCCAGTCTTGAACTGCTTCTCCCTGAAGATGACTAGAGACGATAAATCCACCTTTTTTGCCGCCAGTCATGTCATATTTGAGCTTATCCTCAGCAGGAAGAGCAAAGAAGTCTCTAGCGAGACGAGTCATCTCGGAAACAAGCTTAGTATCAACACCATGATCAACGACCTGGAAAACTCCCCAATCCTCACAAGCGTCGACGATCTTCTTACAAATCTCGTCCCTCTTTCCTCCGAGATCGTCGATTCCAGCAAGCGAGATCACCGGAATGTCGTTGCTGAATTGGTTGTAAGCAACCTTAGGCCTTTCGTCCTCGTCACGGACGAAGCTGGTGTTGAGCGTTGTCTCCTCTGACAGTGCAGTCAGAGTCCTGGGTTTAGTTGAGGCAGCCATTGATGAATGATTTGTTGTTTAGAGTTGGGTGACTTCGAAGTGTTGGATGAATTGACTCTAAATAGGCGCCGGTGGAGAGTAAAGAGGAGGATGGGTAAGATTGTATCTACCTACCCTTCGCAAGAAGTCGTGGCAACTACCCGCTCATTTGTTTCTCACCTTCTAG

***F3’H***

TGATTGTGAATAAACACATTGTGTTTCGTATTGATTCATCTCGGGCCCAATTAAACACAACTAAGTAAGTAGGAAAAGCATCTCAATCATTAGATTTTCTTCTTCTTTCATTTGGGTACACGTAAAAGAGACCGGGATTCAGGATAACATTTTTATTAAGTGTCACGACATATAAGTTCCTAGACTTCCCGATCATTAAGCTTGACGAGGAATCATCGCCAACCAAGAAAAAGAAGCATACAAAAAAAGAGGGGGAAAAAAACATTCATCTAAGAGAAATCAAGAAACCTGGGACTCACACATTGTAAGCATGGGAAGGCAGCCTATGCTTAGGATAAACCATCAAAGGAACCTTACGTTGCAACGTAAGTCCATAAGCTTCTTCCATATCCAACTTCTCCTGCGTCTGACCATCCGCGAGCTCCCAATCAAACGCGTGAACGAGTGTCGCCGTAAGAAACTGGACCATCCTAAGTCCAAGACTCATACCAGCACAAATGCGTCGACCCGCCCCAAACGGGATAACCTCGAAATCATTCCCTCTAACATCCGCACCCGGCCTCTCTCCGCCCGGTAAGAACCTCTCGGGTCGGAACTCGAGTGGGCTCTCCCAAGCGTCGGGATCTCGAGCAATGGCCCACACGTTAACGAGGAGGGTGGCGTTCTTGGGGATGTTGTAGCCGTTTATCTCGCAGCTTTCAGCGGCCATTCTCGGCAGGGAGAGTGGAGTTGACGGATGAAGACGGAAAACCTCTTTCATTACAGCTTGGAAGTATGTTAGGCTTGAAAGATCCGACTCCGTTACGACACGATCGCGGCCCACGACCGTGTCTAGCTCCTCTTGTAGCTTGGCCATGATCTTCGGGTGCCGGATTAGTTCCGCTATTGCCCATTCTGTTGTGCTTGATGAAGTGTCTGTTCCTGCTGTGAACAAATCCAAGAGCAGAGCCTTGATTTCAGTATCAGTCAGCTTGCCGCCTTCGCCGTCACAGTTATCCTTCAAAGAGATCAAAAGGCTAAGCAAATCAGAGTGTTTCTTGCTCTTATTAGACTCAACCATAACCTTATGCTCCTCCAAAATCTGTCCGATAAAGCTGTCAAATCTCTTGTGAAGCTTCTTCATCTTCCCAGCAATGCCTTGGAGATCAAGCGAGTCAAGGATAGGGACAAAGTCACCAATGTTAAACTCTCCGGCCAGAACCATGAGCTCCACAACCATATCCTTGAACTCATCGGCTTTCGGGTCCGCCTTTCCGGAGCCGTCTCCGAACACTCTCCGGCCAAGCATCACTCTTCCTAGGGCATTGGTGGTGCACACGTTAAGGAGTTGCCCTAATTGTACAGCGTTCTTCCCGGCCTTGGATAATGCCCTTGTCAAAATCCCTACCTCCTCCTCACGAACAAATCGGAAGTCCTCCAAGGCCTTGCCGGAGAAAAGATGAACTTGACAGATCTTCCTAAGCATACGCCAGCGGGGACCGTAGGGGGCAAAAACCAAATCCTGGTAATTATAAGCAATATGCTTGGCGCCGGAGTTGGGTGGCCGGGAAGAGAAGTTAGCATCGTGGGTCTTAAGAAATTGGGAGGCCACGGAGGCGGAGGCGGCGATAATGACGTGAACGGAACCCAATCGGAGATGCATGAGAGGGCCGTAGACCTTGGCCAGCGCTGCTAGGGAGTGGTGGGGGACGGCGCCCATGTGTGGGAGGTTTCCGATGATGGGCCATGGTTTGGGGCCCGGTGGGAGGCGGGCGGGCCCCCGGTTGAAGAGTGTGTTGTAGAGGATGTAGAGGGAGAGGAGGGCGGTGAGAGAGTAGATGGTGAGTTGAAGAGTTGATAGGGATTGGATGAAAGGGTGGTTCAGGAGAGAGTTTATTTCCATTTTTTTGGGGATTGTTGAGTTAGCACTTAGTAAGGAAGTGAGATGAGAGTGTAAGCAATGCTTGGGATTTATAATATTACACTCTATGATGCATTATTTTAGGAAGAAAAGAGGTTGGTGGCTGCCTGTCTTGTGTTGGATGGATCTCTCATAGATATATGTACACACACATAGAGAGGAGTTAGGTTAGGATAATAACCCCTCTTAGTCTTCAATCGATTTGGAATTTCTCTCATAGATATATGTACACACACATAGAGAGGAGTTAGGTTAGGATAATAACCCCTCTTAGTCTTCAATCGATTTGGAATT

***F3’5’H***

GGTAACTATTCCAATTTAAAAATGTTTATACAAACAGAAAGCCATATATATAAACTCTCACTAAGAAGTGGACTTTATGATTGATTTAGGAAAATAAAAACATAACCAAAGCCCAAATTAGACATTGATCCCAATGTTTCCAACTTTCTTGAATCATCAATCAACATAAGCACATTGGTCCAATCTTGGTGTTGCAAAAGCCTCAAGCCTATCAGCTTTCCCAAGCACCAATCCAAATTCTTCATCTAACCCAACATCAACACCATATGGTAACCTCCAGTCAAATGAATGAACCAAAGTACCTATAATAAATTGAACCATAGATAACCCCATTCTGATACCAACACAAATCCTTCTACCGGACCCGAACGGTATGAGCTCGTAGTCGTTCCCCCATGGCTCGATGACCGACCCTTTCTCGGTCATAAACCTCTCGGGTTTGAACTTGAGTGGGTCCTCCCATATCGAAGGGTCCCTTCCCATGGCCCACATGTTTATGAATAGCCTTGTGTCTTTGGGGATGTGATAGCCGTTTATTTCACATGCTTTGTTTGTCATTCTTGGAAGACTTAGTGGCACTACTGGGTGTCTTCTGAATGTCTCTTTGCATATTGCTTTTAAGTATGGAAGGTTTGGGATGTCGGATTCTTGTACTCGTCGATTCTTGCCTACGACTTGGTCGATTTCTTCTTGCGCTTGCGTTAGGATTGTCGGATTCTTCATCATCTCCGCCATCGCCCATTCGATTGTGCTTGATGATGTGTCTGTTCCGGCTATAAACAAATTCAATAGAAGGGCCTTGTAGTTAACCATCTTAAGCTCAACACCATCAACTCCTCCATTAATCCCCATCACAATATCAAAGAAATCAGGGTTATCCTCACGATCAATAGTCGTCTTCATGTGTTCTTCCAACATCATCGTTATAAGCGCATCAAACTTATCATGTAGCTTCTTCATCTTCCCTTGAATCCCATGCAAATCCATCCACTCAATACAAGGTATAAAATCACCAATATTAAACAATCCAGCCAACCTCATAAGCTCAGTAATCATATTCTTAAAATCATTAGACTCCTCACCACGAGTATCAAACACTCTCCTACTAATACTCTTCTGACCAATCACATTAGCCAATGCACAAGACACCATCTCCAAGATCGGAACCGGGTTCGGGTTTTTCGAAATCTCGGTCACCATGAACCCGACTTCTCGGAATCTCAACGGAGCCCAATCAAGAAACGACTTAGTACCAAACATGTGAAGATTAGCTAGCTTCCTTAACATCTTCCACCTCGGACCGACGTCGGCAAACACCATATCCTCCATGTTGTAGGCAATGTAAGTCGGACCGGCGCCACCCGGCCGGTTTACGAATTGATGATCAAGATTCTTCAAGAAAGTCTTGGCGATTTCAGGACTTGAAGCTACAACAACGTCACATGATCCTAGCTTTAAGTACATTAATGGGCCGTGTTTTTTTGCTAAACGGGTGAGGGAGATATGAGGCATGGCTCCTAAGAGAGGTAGGCAGCCTAGTACCGGCCACCCGGTTGGACCCGGAGGCAAGCGTTTGCGGGTGAAGAGATGATGGATGAGGAGGGAGAGTGGACCTCCGATGAGGAGAATTTGGAGAAGCAAGGGAATCTCCATTTTTGAGTTTGTTTTTGCGTAGGATTTGAAGTGCTTGGTGGGTTTTTGATGGTTAAGAAGATTAGCAAATCTAGTGAAT

***FLS***

GATCGGAGAAGGAGCAACCGGCGAAAACAACCGTCGAGGGCTCGGTCTTGGAGGTTCCGGCAATTGACATATCGGAGGCGGATGAGGAGGTGGTGGAGATGATTTACAAAGCGAGTAGCGAGTGGGGACTCTTCCAAGTGGTGGGCCATGGGATTCCGAGTGACGTCATTTCTCGGCTTCAGACGGTGGGTAAGGAGTTCTTTGAGCTCCCCCAGGAGGAGAAGGAGCGGTACGCCAAAGCCGACGACTCGAAAGACATCGAGGGTTACGGGACGAAGCTTCAGAAGGAGGTCGACGGGAAGAAGGGTTGGGTTGATCAT

***DFR***

GGGAGGAACAATATCACAAATCACTCTAATCACTCTTTCTCATTTCTTCCCCGGCCTTTTCTCACCAAAACGACGTCGCACCATGGTTGCTGAGGGAGAGATCGTCTGTGTCACCGGCGCTTCCGGCTTCGTTGGCTCATGGCTCGTCATGAGGCTCCTCGAACACGGCTACGTTGTCCGTGCCACCGTCAGGGATCCAAGCAACATGAAGAAAGTGAAGCACTTGTTGGATTTGCCCAAGTCGAAGACGAATTTGAGCCTCTGGAAAGCCGATCTCAGTGAAGAAGGAAGCTTTGACGAAGCAATTCAAGGCTGCGCTGGTGTTTTCCATGTTGCGACTCCCATGGATTTCGAGTCCAAGGATCCTGAGAATGAGGTGATTAAGCCAACCATCAATGGTATGCTGGACATCATGAAAGCATGCCTGAAGGCGAATGTGCGGAAATTGGTGTTCACATCTTCAGCCGGAACAGTCAACGTTGAAGAGAAACAAAAGCCTGTGTACGATGAGACTTGCTGGAGTGACGTTGACTTCTGCCGAAGAGTTAAGATGACTGGCTGGATGTACTTCGTATCCAAGACATTGGCAGAACAAGCAGCTTGGAAATTTGCTGAGGAAAACAACATGGATTTCATTAGCATTATCCCAACTCTCGTTGTCGGCCCTTTCATTATGCCAAGTTTCCCTCCAAGTCTCATCACAGCCCTCTCCCCAATCACAAGAACTGAGGGTCACTACACAATCATAAAACAATGTCAGTACGTACACTTGGACGATTTATGCATGTCTCACATTTACCTCTATGAGAAGGCTGGCTCGAAAGGACGTTACGTTTGTTCTTCCCACAATGCTACCATTTATGACCTCGGAAAAATGCTTCGAAACAAGTATCCCGAGTACAATGTCCCTACCAAGTTTAAGGATTTCGATGAGAACATGGAAGCCGTGTCGTTCTCATCGAAGAAGCTGACCGATGAAGGGTTCGAGTTCAAGTATAGCTTGGAGGACATGTTTGTTGGTGCTGTGGAGACTTGTAGGGAGAAGGGCTTGCTTCCCAAAACCTTTGAGGAGATTGAGAAGAACCATGTAAATGGTAATGGCCATTGATTAGTTTTACTTTTGTGAGAATGGAAGGTGTTTGGAGGGATGTATTTTTATGTCTTACTATTATTGTCTTTTATCTCATATAAGCTTTTCATCAATAAATAAGTTTGGTGCTTTTCGCTTCGTTAAAAAAAAAAAAAAAAAAAAAAG

***ANR***

GGAACCTACTCAAAATCTCTTAGAGTAGGGAATATAAAACAACTTAAGACCAGCGTTGTGCTGCACAATAACACACTTTCACACTAATGCTTAGTTCTTTAGATATATAACCAAGTTAATGGCAGTTCCATAATTTGATGGTCGATTACAGAGGATATTCATATGAGCAAAGCAACTTATATAATCATCTCAATCCGGAAAACTAGGATTGAGGATACATATTAGCATTATTCAATGGAAGTATTACATAACTAGCCAAAGCACGAGTAGAGTATTCAACGTACATGGAAGAGCTGCATTGTAACATAAAGTGGAACTAAGACACTCGACATTTGTTCCAGGTGTAGCCCTCTGCTCTTTCTAGTTCTTTAGCATACCAACTTCCTTCAGGTACTCCACAGTCTCATCGTAGATCTGTTCGATTCCGTACTTGAAGCTGAACCCCTCTTTGGTAAGCTTCTCAGAAGAGAGCACCAGCTTTGTTTTCGATGGGAAATCACCAAAGTCTGTGGGGACTTTGTACTGGGGATATCTCTTGCTCAAGAACTTGGCGAGCTCAGGGACGCTCGTGTTGACAGCACAGCAAATGTAACGGCCGGAGGCTGACTCCTTCTCCGCTACAAAGATGTGAGCTCTGCAAACATCCTCCACGTGAGAAATGGAGATAGAACCAGACAGCATTTGCATACCCTTCAATGCATTTTGTAAGAAATCGTTTCCACTTAGCAAGCCAGTGGCGAGTCCAATACTGCTGGGGACATCAGAAGTAATAGAACGGCCGGTCATCAAACTTGGGATAACAGTAACCAGATCAATGTTATGTTCCTCTGCAAATTCCCAAGCTTTCTTTTCCGCAAGCATCTTGGATGCAGGATAGCCCCAAGTTGGAGGCTTTGCATTCGACAAGAACTCAACATCAGTCCAGTTGTCTTCAGTCATGACCAGGCCTGTCCCTTCCAGAGTGTTGATCGACACAGCAGCAGCCGACGATGTCAAAACCACACGCTTCACAGATTTCACTCTTGCACAGGCTTTCAGTACATTCAGCACTCCTTGGATAGCTGGCTTGATCATATCGTTCTCTGGATCTTCAGAAGCAAAGTTGACAGGGGTTGCTACATGAAAGACGAACTCACAACCCTCTATCGGAGCATCAAAGCTCAGCTCATCAGTTAGATCAGCACGAAAGATCTTTACATCACCCAACTTTTGTAATTCTACGAGGTGAGAAACCTTTGTTTGGTTGTCAGGATCTCTAACAGTGGTGTTGACAGAATAGCCCTTCTCAAGCAATAATTTGATAAGAGTAGCGGCGACGAATCCAGACCCGCCAATAACACAGACCTTCTTATCACCAACTTGGGCAGCCATGATTGAGGGATTAGGTCTAAATCCTTCTATTAAATTGTTTGGGTCCTTAGAAAAAATGGAATTTTGAGTTCACCACAGAACACTGAAGCAATCGAAGTGTCACTTCGGGGTATAGGTTTTGAGTAATATATAATGTTTGATAATAAGAAGAAGGTAAGCACGTGTCTAGCAGCACACCTTCCACACCGCTGCAACGCACGGCACCACCAACCAACCCTTAAAACATAAGGCACTTGATAAG

***ANS***

TTTTTTTTTTTTTTTTAAGAAAAGCTGATAATGATTCTTTGAGCAGTTTTTTTTTTATTGAACATCAAGAAGCAGCAGGGTAAACACAATCCAAGCAGGACCCATCATCTAATGACATAATACCTCCCGCCTCTAACATGCAGTATAATGACTACATAAAATGCAACAACTGGAAAAATAATCCACTTTCAGAGACTCCCAAATTATAAAAACACTCAAGTATAGATACTAAATATAGATAGAGAGAGAAGTGATGATCAATCCGATCCACCATAAGTGCAAACATATTCATTTTCAATTTATGACCAAAATACCCTCACTTGTTCAAACCATGCAAATAAGCTTTTTTATTCCTTGGAAGCTGGGGCCTGCAGTTCTTGAGTCTTCCTGAACAGCTTATACTCAATGTGCTGAGCAAAGGTACGCGGCGGAAACTCTGCCGGCTCACTCTCCGACACAAGCTCCGGCAGAGGCTTAAGGATGATCGAATGCTTTGGCGGCTCGCAGAAAACTGCCCACGAAATCCTAACCTTCTCCTTGTTCACCAACCCCCTATGAAGAATACTCTTGTACTTCCCGTTACTCAGGATAGTCAAAGTATCACCAATATGCATGATGATCGAGTTCGGAACACATTTCGCAGTGACCCATTTGCCCTCGTAGAACAGCTGCAGTCCGGGCACCATGTTGTGGAGAATGAAGGTCAAAGCACTCACATCAGTGTGTGCCTCCACTCCAAGCGCCAGCTCCGGCTGGGGGCATTTTGGGTAATAATTGATCTTCATTTGTAGAAGAAGCTCCTCTATCCCTCCTACTTCCTTCTCTAGCCTGTCTTCTTCTAGCCCGAGCGCCAGCGACAGAGCCGACATGATCTTCGTCGCCAAGCTCCTCAATTCCTTCGCGTACTCGCTCGTCGACGGAATGTAGTCAGAAGGAGTCTGAGGCCAAACGGACAAGTCTCTCTTGTCTTCGGGGTAGGCAAGGTGAAAAAAGTAATCTTCCCACTCAAGTTGACCGCTAGCATTGTTGGCGAGCTTGCTGCCATAGCCTTGGATCTTACCGCTACCTTGATCATTGGCATATTTCTCCTTCTCCTCAATAGGGAGTTCGAAGAAGGTGGCACCGGCATTCTTGACGCGGGTAGTGAGCTCGTCGGGGATGCCATGGTTGACTAAATGCATGACACCCCATTCGGTGGCGGCCTTCTTGAGTTCCTCAATAGCCTTAGCCCTGACCTCCTTGTCCTCGGACGCTATGTCCTTGATGTCGATGGTCGGGACTTGGGGACCTTCCTTCTTCTCTTCCTCGAACACGTTTCCGATGCTCGTGAGCTCCTCTTCCGGCCTAACATACTCCTTCGGGATCGATTGGATCCCGCTGCTCGCCAAGCTCTCGACTCTTGAAGAAGCGATGGAAACCATTTCAATTATCTAGTTTTATTTTGAAGGACACAAATATGACAAATAGATTGAATTTGTAGTTGAATGGTGTTTGATTC

***LAR***

GTCACTTGCCAAGCTCTCTCTTCTCTCCCAAAACGAGCGAGATTGTTAAGTGAGTCGATCTTCCAGCAAGTCAATATGACTGTTGCCGTGACCGCCATCCCTGAGTCAAAGTGCCGGACCTTGGTGGCCGGAGCAACCGGGTTCATTGGCCGGTTCGTTACGGAATCGAGCTTGGAATCGGAGCGACCCACTTTCATCCTGGTTCGACCCGGGCCCATTTCCCCTTCCAAGACTAAAATCATCAAAGCCCTAGAAGACAAAGGTGCCATAATTGTTCAGGGACTTATAAATAACAAAGAACGAATGGAGAAGATACTCAGAGAAAACGAGATTAACGTTGTCATATCCGCCGTCGGAGGAGGCAACATACTAGACCAGATTCCTCTTGTCCATGCCATCAAATCGGTCCCTAGCGTTAAGAGGTTTCTGGCGTCGGAATTTGGGCACGACGTGGACAGGGCGAACCCGGTAGAGCCGGGGCTGACGATGTACTTGGAGAAGCGAGCGGTGCGACGGGCGATAGAGGAGGCCGGGGTGCCATACACGCACATATGTTGCAATTCCATTGCGTCGTGGCCGTACTATGATAACACTCACCCCTCGGAGGTCACGCCTCCTATGGACCGTTTCCAAATCTATGGAGATGGAAACGTTAAGGCTTACTTTGTCGCCGGCTCTGATATCGGTAAGTTCACAATCAAGACTCTGGAAGACCCACGAACGTTGAACAAGATGGTTCATTTCCGGCCGCCTTCCAACTACCTAACAATCAATGAGCTGGCAAGCCTTTGGGAGAAGAAGATCGGTAAGACCCTGCCACGTGTCACGGTCACCGAGGACGACTTGCTGGACTTAGCTGCAGAGAATCGTATCCCAGAGAGTATAGTGGCGTCATTCACTCATGACATATTCATAAAGGGATGCCAGGTGGATTTTGCAGTTGACGGTCCGAATGAGGTTGAGATCGAGAAGCTTTACCCAAAGGATAAGTACATAACCATAGATGAGTGCTTTGAAGAGTTTGTTATTACTAGTAACAACAACAAAGAGATTATAGAAGAGGTGGTTGTCACTGAAGCTTTTGATGATGAAATTGGAAACAAGAAACAGAGCAATAAGAGGAATGTTGAAAATGAAGAGGATGCAAGTGGAAACAAGAAACAGAGCAGCATGAACAAAATTACTAGTACTGCTGCTGCTGCCAAATCAAGCCATGTTGTTGAGGCCTTACCAGTCCCTGCAGTGTGCTGAGATGTACTGGATCACTACCTCTCTCTTTCTCTTTCTACTAAGATTGCTGTCCTGCCCCAAATAATCTTTATCGGCATAAATCCACTATTTAGGTTTTTACTTGTTTCTGTGTTGCTCAATGAGGTTAAATTTTACTCTTTTTTTTTTAATTTATTTTATCCCCATAGAGCTTAGAAATTAAGAATGTGTTTGTTGATGCCATTTTTACCCACTTTTGAGGGGGACAATATTGTAATGTCTACATATTCATGGATTTCCTAATGTATTCACCAATAAAGCACCAAGCTAAGCGAGCTATCTTCTTGAGTTATTCTCCTACAAAAAA
