# Supplementary material for: Transcriptome Analysis of Differentially Expressed Genes Involved in Proanthocyanidin Accumulation in the Rhizomes of Fagopyrum dibotrys and an Irradiation-Induced Mutant
Source: Front Physiol. 2016 Mar 18;7:100. doi: 10.3389/fphys.2016.00100 (PMC4796566; doi:10.3389/fphys.2016.00100)
Supplement: Supplementary file 2 [file Presentation2.ZIP › Supplementary Material/Supplementary Material.docx]

Supplementary Material

Transcriptome analysis of differentially expressed genes involved in proanthocyanidin accumulation in the rhizomes of ***Fagopyrum dibotrys*** and an irradiation-induced mutant

Caixia Chen, Ailian Li^*^

*** Correspondence:** Ailian Li, Cultivation Center, Institute of Medicinal Plant Development, Chinese Academy of Medical Science & Peking Union Medical College, Beijing, 100193, China

alli@implad.ac.cn

Supplementary Tables

Additional file 1. Primer sequences used for qRT-PCR.

Additional file 2. Gene function classification based on Gene Ontology.

Additional file 3. Gene function classification based on KEGG

Additional file 4. The top 100 unigenes with the highest levels of gene expression in RM_R and CK_R separately.

Additional file 5. The 204 clearly upregulated genes and 297 markedly downregulated genes .

Additional file 6. GO enrichment analysis of the differentially expressed genes (DEGs).

Additional file 7. The statistical enrichment of DEGs in the KEGG pathways.

Additional file 8. A total of 452 unique sequences encoded putative TFs detected in *F. dibotrys* roots.

Additional file 9. Sequence information of PAs biosynthetic genes in *F. dibotrys* roots*.*
